# Surface plasmon-enhanced photo-driven CO_2_ hydrogenation by hydroxy-terminated nickel nitride nanosheets

**DOI:** 10.1038/s41467-023-38235-9

**Published:** 2023-05-03

**Authors:** Saideep Singh, Rishi Verma, Nidhi Kaul, Jacinto Sa, Ajinkya Punjal, Shriganesh Prabhu, Vivek Polshettiwar

**Affiliations:** 1 Department of Chemical Sciences, Tata Institute of Fundamental Research, Mumbai, India; 2 Department of Chemistry-Ångström Laboratory, Uppsala University, Uppsala, Sweden; 3 Department of Condensed Matter Physics and Materials Science, Tata Institute of Fundamental Research, Mumbai, India

**Keywords:** Energy harvesting, Energy, Photocatalysis, Chemical physics

## Abstract

The majority of visible light-active plasmonic catalysts are often limited to Au, Ag, Cu, Al, etc., which have considerations in terms of costs, accessibility, and instability. Here, we show hydroxy-terminated nickel nitride (Ni_3_N) nanosheets as an alternative to these metals. The Ni_3_N nanosheets catalyze CO_2_ hydrogenation with a high CO production rate (1212 mmol g^−1^ h^−1^) and selectivity (99%) using visible light. Reaction rate shows super-linear power law dependence on the light intensity, while quantum efficiencies increase with an increase in light intensity and reaction temperature. The transient absorption experiments reveal that the hydroxyl groups increase the number of hot electrons available for photocatalysis. The in situ diffuse reflectance infrared Fourier transform spectroscopy shows that the CO_2_ hydrogenation proceeds via the direct dissociation pathway. The excellent photocatalytic performance of these Ni_3_N nanosheets (without co-catalysts or sacrificial agents) is suggestive of the use of metal nitrides instead of conventional plasmonic metal nanoparticles.

## Abstract (web summary)

Plasmonic catalysts are often limited to Au, Ag, Cu, Al, etc. Now, hydroxy-terminated nickel nitride nanosheets are reported to be an alternative material for CO_2_ hydrogenation to CO using solar energy without co-catalysts or sacrificial agents.

## Introduction

The photocatalytic conversion of CO_2_ into valuable solar fuels and chemicals is an appealing way to recycle carbon while addressing global warming and the energy issue^1–3^. Plasmonic nanocatalysts, through their localized surface plasmon resonance (LSPR) process, generate localized electric fields, hot carriers, and heat, providing unique capabilities for sustainable CO_2_ reduction catalysts^2–14^. Visible light-active plasmonic catalysts are limited to Au, Ag, Cu, and Al, which have considerations in terms of costs, accessibility, and instability, in particular with Cu and Al^2,4^. This raises a pertinent question: is it possible to replace metal nanoparticles with an alternative and stable plasmonic material, one capable of performing a photocatalytic CO_2_ reduction reaction?

Nickel nitride (Ni_3_N) nanosheets have been hypothesized to possess plasmonic abilities in the visible domain and show good electrochemical properties^15–25^. Carrier concentration and electric conductivity are also high in Ni_3_N nanosheets, which is a prerequisite for plasmonic activity. However, it remains to be answered if that can be harnessed to photocatalyzed CO_2_ reduction without external electrical bias, heat, co-catalysts, or sacrificial agents.

In this work, we have synthesized nanosheets of Ni_3_N by a solvothermal process. The synthesized Ni_3_N nanosheets were found to be hydroxy-terminated and have good light-harvesting capability. The nanosheets were able to catalyze CO_2_ hydrogenation reaction using solar energy with a high CO production rate of 1212 mmol g^−1^ h^−1^ in the flow conditions, with almost 99% CO selectivity (Supplementary Table S1). The role of plasmon excitation was studied by: (1) light intensity-dependent production rate, (2) wavelength-dependent production rate, (3) kinetic isotope effect (KIE), (4) light intensity-dependent photocatalytic quantum efficiencies, (5) competitive CO_2_ hydrogenation in the presence of electron quencher, methyl-p-benzoquinone (MBQ), and (6) nanosecond transient absorption spectroscopy. The molecular reaction mechanism of CO_2_ hydrogenation was studied by in situ diffuse reflectance infrared Fourier transform spectroscopy (DRIFTS).

## Results and discussion

### Synthesis and characterization of Ni_3_N nanosheets

**Fig. 1:**
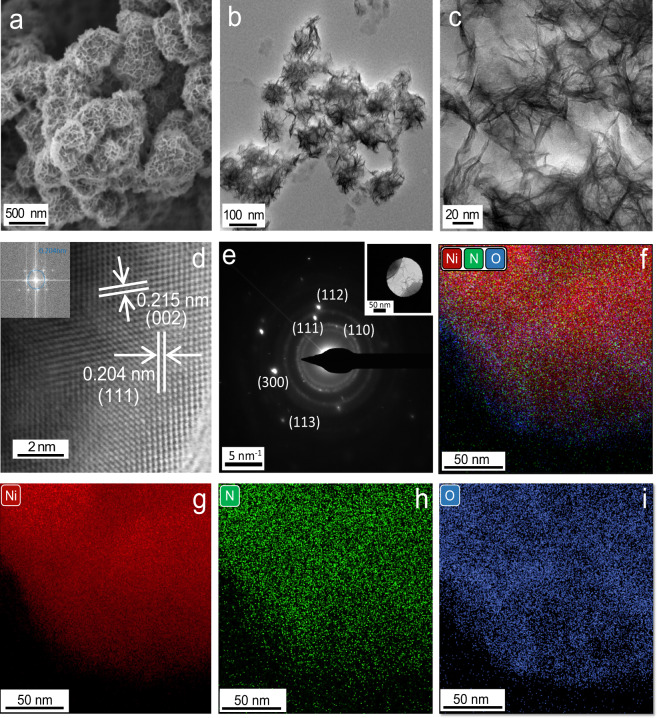
Electron microscopy analysis of Ni_3_N nanosheets. **a** SEM image, **b**, **c** TEM images, **d** HAADF-STEM image (Inset: corresponding diffraction pattern), **e** SAED pattern (Inset: TEM image of the corresponding area), (**f**–**i**) STEM-EDS elemental mapping of Ni_3_N nanosheets.

For the nickel nitride nanosheets synthesis, nickel acetylacetonate (nickel precursor), Li_3_N (nitrogen precursor), ethylenediamine, and o-xylene were heated in the autoclave at 270 °C for 20 h, and the isolated product was washed with ethanol and water (Supplementary Fig. S1 and Methods section for detailed experimental). Scanning electron microscopy (SEM) analysis of Ni_3_N showed the formation of aggregates made up of self-assembled nanosheets (Fig. 1a, Supplementary Figs. S3a, b and S5a, b). Transmission electron microscopy (TEM) analysis showed the thin sheet-like morphology of each of these Ni_3_N nanosheets (Fig. 1b, c, Supplementary Figs. S3c–f, S4, S5c–f). The dark contrast between the edges and the center was due to the presence of crumbled sheets of different thicknesses. High-angle annular dark-field scanning transmission electron microscopy (HAADF-STEM) revealed that the lattice plane matches the (111) plane of the Ni_3_N lattice (Fig. 1d, Supplementary Figs. S4, S5f). The high crystallinity of nanosheets having different lattice planes of Ni_3_N was also observed by the selected area electron diffraction (SAED) pattern (Fig. 1e). Scanning transmission electron microscopy (STEM) with energy-dispersive X-ray spectroscopy (EDS) elemental mapping indicated the uniform distribution of Ni and N over the entire nanosheets along with the uniform distribution of O (Fig. 1f–i). The atomic percentage of Ni, N, and O was found to be 63.5 ± 4.7, 18.2 ± 5.3, and 18.3 ± 1.2, respectively, in SEM-EDS (Supplementary Fig. S6). Atomic force microscopy was used to measure the thickness of Ni_3_N nanosheets and the average thickness of the nanosheets was around 8 nm after taking the height profile from multiple points (Supplementary Fig. S7).

**Fig. 2:**
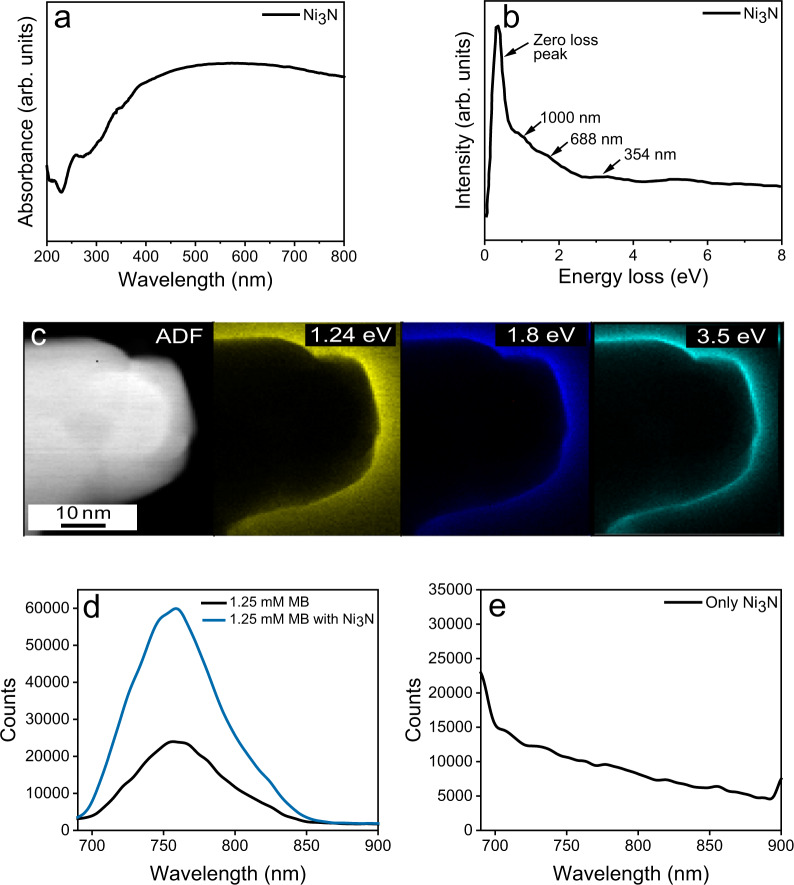
Optical characterization of Ni_3_N nanosheets. **a** UV-DRS spectrum, **b** EELS spectrum of Ni_3_N nanosheets, **c** EELS plasmonic mapping of Ni_3_N nanosheets for different electron energy losses, **d** Emission enhancement of the methylene blue (MB) (1.25 mM) in the presence of the Ni_3_N nanosheets dispersion (0.5 mg mL^−1^ Ni_3_N in water) upon 630 nm excitation; **e** Photoluminescence of Ni_3_N nanosheets dispersion upon 630 nm excitation.

The Ni_3_N nanosheets showed broadband light absorption from the visible to near-infrared (NIR) region due to plasmonic excitation and scattering (Fig. 2a). The electron energy loss spectroscopy (EELS) of Ni_3_N sheets showed zero loss peak at 0.57 eV while four less intense broad peaks between 1–4 eV (Fig. 2b), which were attributed to plasmonic excitation of Ni_3_N nanosheets. To visualize the spatial distribution of localized surface plasmon modes, we then performed the EELS mapping on the Ni_3_N nanosheets (Fig. 2c). The losses around the edges of the nanosheets correlate well with the observed absorption profile of the Ni_3_N nanosheets. The LSPR excitation in nanomaterials is always accompanied by an elevated electric field in close proximity to the nanomaterial’s surface. This high electric field for Ni_3_N nanosheets was estimated using finite-difference time-domain (FDTD) simulation. The electric field near the edges of the nanosheets was enhanced, and the maximum enhancement was found to be six times at the corners of the sheets (Supplementary Fig. S8).

Reactant molecules that are in close proximity to the elevated electric field of the nanosheets will have more probability of photo-excitation^26,27^. To further investigate the plasmonic nature of Ni_3_N nanosheets, we carried out photoluminescence (PL) studies of methylene blue (MB) dye in the presence and absence of Ni_3_N nanosheets. After excitation by a 630 nm laser, pure MB emits a broadband emission centered around 760 nm (Fig. 2d). Notably, in the presence of Ni_3_N nanosheets, this MB emission increased by a factor of 2.6. (Fig. 2d). The high electric field of the Ni_3_N nanosheets causes more MB dye molecules to be excited, resulting in higher emissions after the molecules are relaxed to the ground state^26,27^. Since Ni_3_N nanosheets did not show a strong PL, the possibility of its contribution to emission spectra of MB adsorbed on its surface was minimal (Fig. 2e). Thus, EELS, plasmonic mapping, electric field enhancement by FDTD, and PL enhancement confirm that Ni_3_N nanosheets are plasmonic.

**Fig. 3:**
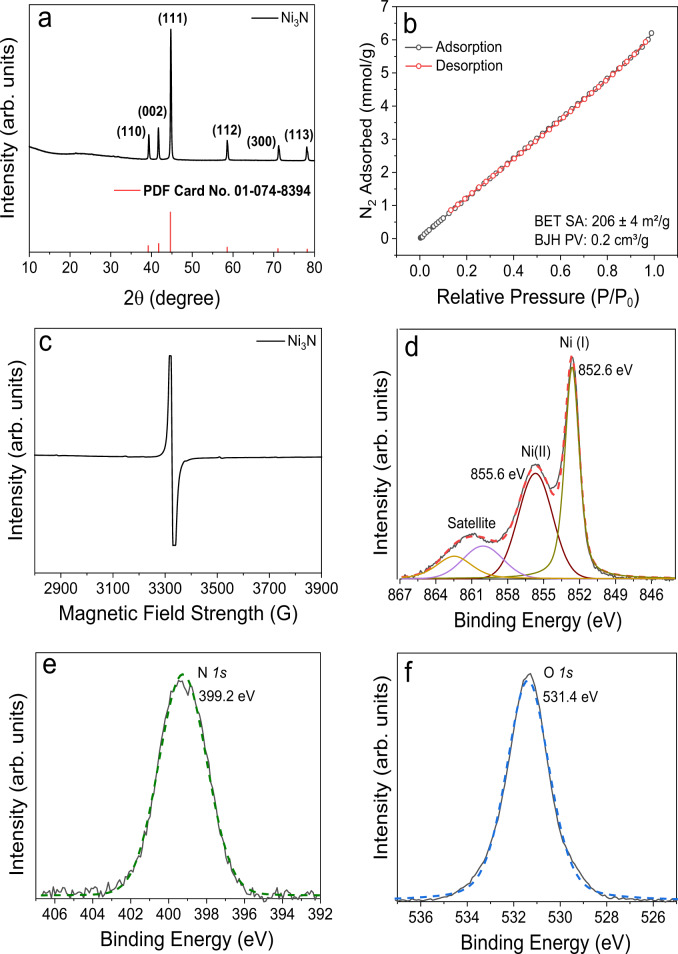
Characterization of Ni_3_N nanosheets. **a** PXRD pattern, **b** N_2_ sorption isotherm, **c** EPR spectrum, and XPS spectra expanded in the region of **d** Ni 2p_3/2_, **e** N 1s, and **f** O 1s, of Ni_3_N nanosheets.

Powder X-ray diffraction (PXRD) patterns of Ni_3_N nanosheets was consistent with that of hexagonal Ni_3_N (PDF: 01-074-8394) (Fig. 3a). The surface area of the Ni_3_N nanosheets was 206 ± 4 m^2^ g^−1^ by N_2_ sorption analysis (Fig. 3b). The Ni^+1^(d^9^ system) electronic state of Ni in Ni_3_N nanosheets was confirmed by the electron paramagnetic resonance (EPR) spectroscopy^28^ (Fig. 3c). The X-ray photoelectron spectroscopy (XPS) analysis of Ni_3_N nanosheets showed peaks located at 852.6 eV and 855.7 eV of Ni*2p*_3/2_ were assigned to Ni(I) and Ni(II), respectively (Fig. 3d)^21^. In the N*1s* spectra, a single peak with a binding energy of 399.2 eV corresponds to nitride-type nitrogen bonded to nickel was observed (Fig. 3e)^21^. The peak of 531.4 eV in O*1s* corroborated an assignment to the surface OH bonded with nickel (Fig. 3f)^22–24^. Temperature programmed reaction (TPReaction) of Ni_3_N nanosheets (monitored by mass spectrometer) indicated stepwise transformation; step-1: loss of strongly bound water between 120 and 240 °C; step-2: dehydration of terminal nickel hydroxide to nickel oxide between 240 and 325 °C, and step-3: further degradation of Ni_3_N sheets into nickel, N_2_, and H_2_ between 325 and 475 °C (Supplementary Fig. S9) Thermogravimetric analysis (TGA) under argon also indicated the same pattern (Supplementary Fig. S9g).

The electrical conductivity of hydroxy-terminated Ni_3_N nanosheets was found to be 6.25 × 10^3 ^S m^−1^ at 300 K, indicating an intrinsic metallic state of Ni_3_N nanosheets (Supplementary Fig. S10)^17^. The plasmonic excitation after decay generates hot charge carriers, and we investigated these charge carrier generations by measuring the photocurrent from a pellet of Ni_3_N nanosheets in a light on-off cycle (Supplementary Fig. S11). Because of its conducting nature, the Ni_3_N carries a current of 0.296 mA in the dark under an external bias of 100 mV^29^; however, as soon as the light is turned on, the magnitude of the current quickly increases to 0.304 mA. The plasmonic photocurrent showed two components^29^, first a rapid increase in the current corresponding to photoexcited carriers and second slow component attributed to an increase in the resistance of Ni_3_N nanosheets due to their photothermal heating (Supplementary Fig. S11). This indicated the fast generation of excited charge carriers under light excitation of Ni_3_N nanosheets. Since the generation of charge carriers also affects the material’s work function, we measured the change in the work function of Ni_3_N nanosheets using Kelvin probe force microscopy (KPFM) in dark and light^30^. The work function of Ni_3_N nanosheets decreased from 4.585 to 4.579 eV when excited by light (Supplementary Fig. S12). This was due to the generation of charge carriers following plasmonic excitation, which fills the higher energy levels, resulting in a decrease in work function. The fast photocurrent response and decrease in the work function demonstrated the generation of charge carriers in Ni_3_N nanosheets after plasmonic excitation.

### Photocatalytic CO_2_ hydrogenation using Ni_3_N nanosheets

**Fig. 4:**
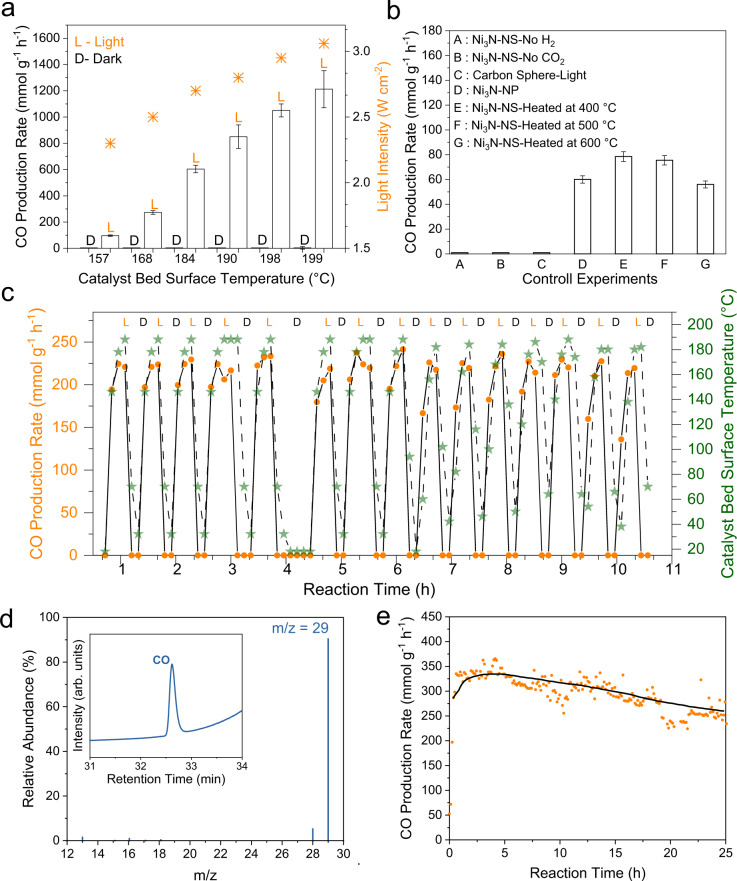
Photocatalytic CO_2_ hydrogenation using Ni_3_N nanosheets. **a** Production rate of CO in light at various intensities and dark at different temperatures, **b** control experiments at 2.5 W cm^−2^. No external heating was used; **c** Production rate and catalyst bed surface temperature during successive light ON and OFF conditions at 2.5 W cm^−2^, **d** Mass spectra of ^13^CO, obtained using labeled ^13^CO_2_ as feed; **e** Long-term stability study under the flow condition using light (at 2.5 W cm^−2^) without external heating. Reaction conditions: H_2_ (4 mL min^−1^), CO_2_ (73 mL min^−1^), xenon lamp (400–1100 nm). Error bars: calculated from data of at least three repeated experiments. NP-nanoparticles, NS-nanosheets. Detailed calculations are given in Supplementary Data 1.

The broadband light-harvesting ability of these Ni_3_N nanosheets and their plasmonic nature motivated the investigation of their potential efficacy for photocatalytic CO_2_ hydrogenation (Fig. 4). The CO_2_ hydrogenation was carried out in a flow reactor with a quartz window for light irradiation using a xenon lamp (Supplementary Fig. S2). The Ni_3_N nanosheets powder was placed in a porous alumina crucible equipped with a thermocouple to monitor the catalyst bed surface temperature. A thin catalyst bed allowed for irradiation throughout. Catalysis was performed in the presence of light (wavelength: 400–1100 nm) without any external heating with a CO_2_ and H_2_ flow of 73 mL min^−1^ and 4 mL min^−1^, respectively (Supplementary Fig. S2). The product formation was analyzed and quantified with time using online micro-gas chromatography (micro-GC).

The optimal total reactant (CO_2_ and H_2_) flow and optimal ratio of CO_2_:H_2_ for CO production were found to be 77 mL min^−1^ and 20:1, respectively (Supplementary Fig. S13). Photocatalytic CO_2_ hydrogenation was then carried out at different light intensities (without any external heating) as well as in the dark with external heating (Fig. 4a). The catalyst bed surface temperature (Ts) was measured by a thin thermocouple inserted directly into the catalyst’s powder bed (Supplementary Fig. S14). The catalyst bed surface temperature was also measured by a thermal IR camera (Supplementary Fig. S15). While we cannot detect the temperature of ultrashort-lived hot spots, the combined measurement approach adopted here reasonably estimated the average surface temperature. An excellent CO production rate of 1212 mmol g^−1^ h^−1^ with almost 99% CO selectivity was achieved at 3.06 W cm^−2^ light intensity (Supplementary Data 1), and Ts was 199 °C (no external heating).

To further confirm the product’s selectivity, the CO_2_ hydrogenation reaction was also monitored using a mass spectrometer (MS). Only CO ions were detected, and no ions of CH_4_ were detected in MS (Supplementary Fig. S16). Notably, when CO_2_ hydrogenation was carried out in the dark at various Ts (observed at different light intensities, 157, 168, 184, 190, 198, and 199 °C), using external heating, no product formation was observed (Fig. 4a). Different control experiments were also carried out using only H_2_ or CO_2,_ and no catalytic activity was observed (Fig. 4b). An isotope experiment was conducted using labeled ^13^CO_2_ instead of the ^12^CO_2_ feed gas. The obtained product signal corresponded to ^13^CO (m/z = 29) in the mass spectrum, thus confirming the reaction product origins from the CO_2_ feed gas and not from any carbon impurities (Fig. 4d). When Ni_3_N was replaced by carbon spheres, there was no CO production. Ni_3_N nanoparticles^17,25^ were also investigated to evaluate the role of morphology. They produced only 60 mmol g^−1^ h^−1^ of CO, which was almost an order of magnitude less than nanosheets (Fig. 4b, Supplementary Fig. S17), indicating the role of Ni_3_N nanosheet morphology in photocatalysis. The Ni_3_N nanosheets stability for photocatalytic CO_2_ hydrogenation was studied for 25 h in a continuous flow reactor (Fig. 4e). Notably, the CO production was stable, with a 10% drop in its production rate in 25 h, without any significant changes in Ni_3_N nanosheets properties based on post-catalysis characterizations (Supplementary Fig. S18).

**Fig. 5:**
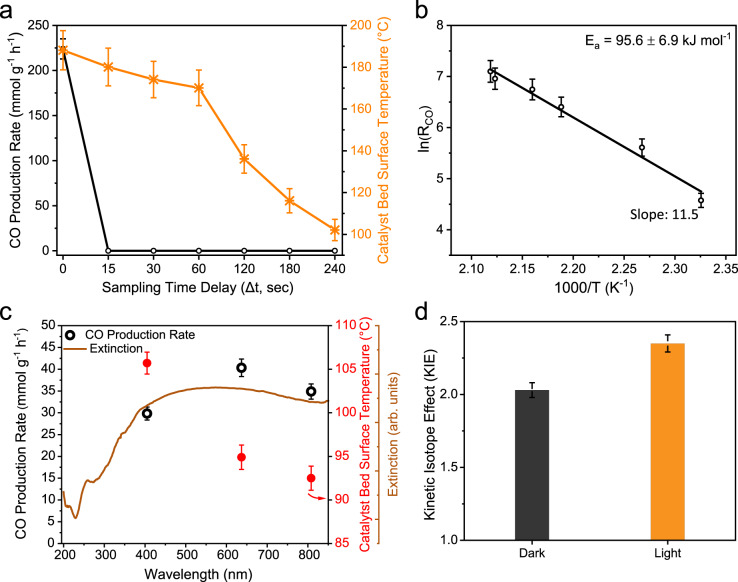
CO production rate wavelength dependence and kinetic isotope effect. CO production rate as a function of the **a** sampling time delay (Δt) after the light was switched off; **b** Arrhenius plot for E_app_ of the photocatalytic CO_2_ hydrogenation in light. The slope of the curve gave activation energy; **c** CO production rate and catalyst bed surface temperature as a function of light wavelength (light intensity at each wavelength was constant); **d** Kinetic isotope effect (KIE) for CO_2_ hydrogenation, measured in dark and light. Error bars: Calculated from data of at least three repeated experiments.

To understand the role of plasmonic non-thermal vs. photothermal effects^10,12,14^, the catalyst performance was measured in successive light (L) and dark (D) modes (Fig. 4c). This study was repeated for 15 successive cycles. The catalyst activity and bed surface temperature (Ts) at various time points after the light was switched off were measured (Fig. 5a). Product sampling time difference (Δt) was defined as the time between switching the light on or off and the GC injection time. We observed that the catalyst became active as soon as the light was switched on, while the surface temperature did not show this fast response, and it took time to reach a saturation value. Notably, the production rate decreased sharply as soon as the light was switched off, whereas the temperature took time to cool down (Fig. 5a). Suppose the thermal effect had been the key driving force in the catalysis reaction, then the CO production rate would not have dropped instantaneously after switching the light off since the surface temperature was nearly the same for some time, even after the light was switched off. These observations indicate the involvement of non-thermal effects, although the role of thermal effects can not be ruled out completely. The Arrhenius plot of the CO production rate under light irradiation showed that the activation energy (E_app_) for the reaction in light was 95.6 ± 6.9 kJ mol^−1^ (Fig. 5b).

To further understand the role of light excitation, we carried out wavelength-dependent catalysis keeping light intensity constant. The CO production rate was found to follow the same trend as the absorption spectrum, indicating the role of plasmon excitation in the CO_2_ hydrogenation reaction (Fig. 5c). We also measured the surface temperature of the catalyst bed under light excitation of different wavelengths; the temperature was maximum (106 °C) at 405 nm and minimum (93 °C) at 808 nm, but the CO production rate remained nearly constant across these wavelengths (Fig. 5c).

We then studied the kinetic isotope effect (KIE) of photocatalytic CO_2_ hydrogenation using D_2_ and H_2_ in light and dark and calculated the reaction rates by counts of CO using a mass spectrometer (Fig. 5d, Supplementary Fig. S19). The KIE in light (2.35) was higher than in the dark (2.03). This enhanced KIE indicated the electron-driven plasmonic CO_2_ hydrogenation^12^. The difference in reaction rate for D_2_ vs. H_2_ was due to different masses of these isotopes, with lighter isotopes experiencing more acceleration under excitation, gaining more vibrational energy, and, thus, in turn, more reaction probability. The large KIE in light also indicates that light-induced local heating of Ni_3_N nanosheets must be playing the role but cannot account for the observed change in KIE.

**Fig. 6:**
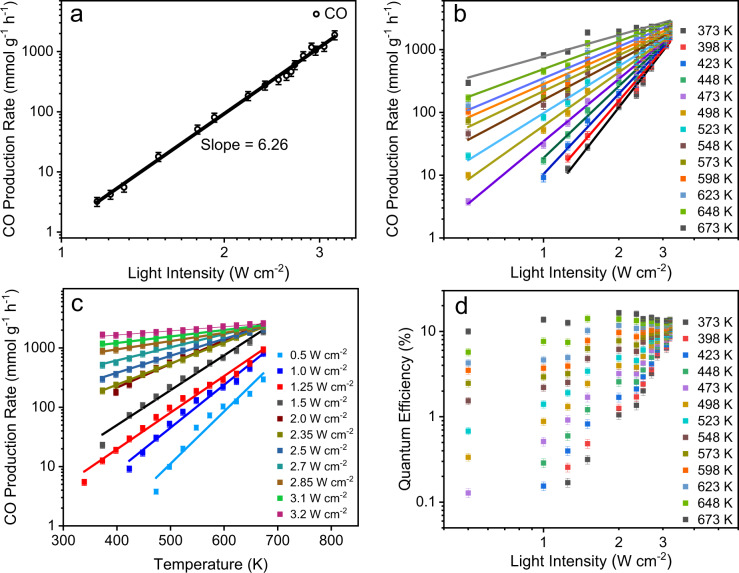
Photocatalytic reaction rate and quantum efficiency as a function of light intensity and reaction temperature. **a** CO production rate (log scale) as a function of light intensity (log scale). The slope gives the power law exponent number; **b** CO production rate (log scale), as a function of light intensity (log scale) at various temperatures; **c** CO photocatalytic rate (log scale) as a function of reaction temperature at various light intensities; and **d** Quantum efficiency (%) (log scale) as a function of intensity (log scale) at various reaction temperatures. Detailed calculations are given in Supplementary Note S1 and Supplementary Data 2. Error bars: calculated from data of at least three repeated experiments.

The involvement of hot electrons in CO_2_ hydrogenation was studied by carrying out the reaction at various light intensities and temperatures (Fig. 6). In the intensity-dependent CO_2_ hydrogenation, the CO productivity showed a super-linear dependency on the intensity with the power law exponent of 6.3 (Rate∝I^n^) (Fig. 6a). The observed intensity dependence is a signature of multi-electron-driven chemical reactions in plasmonic catalysis. Linic and coworkers^12^ observed a similar super-linear dependency of photocatalytic rate on Ag nanocubes, which was attributed to multiple electron transfer processes. Notably, in the case of Ni_3_N nanosheets, we observed the power law exponent of 6.3, as compared to Ag nanocubes’ 3.5 (although for O_2_ dissociation reaction)^12^. This indicates the multiple electron transfer abilities of Ni_3_N nanosheets.

Plasmonic photocatalysts are known to show a positive relationship between reaction temperature and reaction rate in light^12^. Ni_3_N nanosheets also showed a similar positive effect. Figure 6b, c shows that at a constant light intensity, the CO_2_ hydrogenation rate increased with an increase in reaction temperature (by external heating). This positive relationship between light intensity and the reaction temperature impacts the process’s quantum efficiency^12^. We thus calculated the quantum efficiency of this CO_2_ hydrogenation process by dividing the reaction rate by the rate of impinging photons on Ni_3_N nanosheets. At a given light intensity, an increase in reaction temperature resulted in an increase in quantum efficiency (Fig. 6d, Supplementary Note S1 and Supplementary Data 2), a signature of plasmon-assisted photocatalysis.

When we studied Ni_3_N nanosheet’s thermal stability behavior (Supplementary Figs. S9 and S20), it was found that Ni_3_N nanosheets start degrading from 325 °C; hence if Ni_3_N nanosheets’ local surface temperature increases above this temperature during plasmonic catalysis due to local plasmonic heating, nanosheets will degrade and become catalytically inactive. However, Ni_3_N nanosheets were stable for 25 h (Fig. 4e), indicating that surface temperature must be below 325 °C during the plasmonic catalysis. When the CO_2_ hydrogenation was carried out at 400 °C using external heating, the CO production rate of only 80 mmol g^−1^ h^−1^ was observed, indicating degradation of nanosheets during catalysis. To get further insight, we intentionally degraded the Ni_3_N nanosheets by pre-heating them at 400, 500, and 600 °C in the presence of argon (Supplementary Figs. S21b–d and S22). The CO production rate using these degraded nanosheets was significantly reduced to less than 80 mmol g^−1^ h^−1^ in all three cases (Fig. 4b, Supplementary Fig. S21a). Thus, although we cannot completely discard the thermal contribution of plasmonic hot spots to catalysis, these results indicated the involvement of hot electrons and holes.

### Electron transfer studies of Ni_3_N nanosheets

**Fig. 7:**
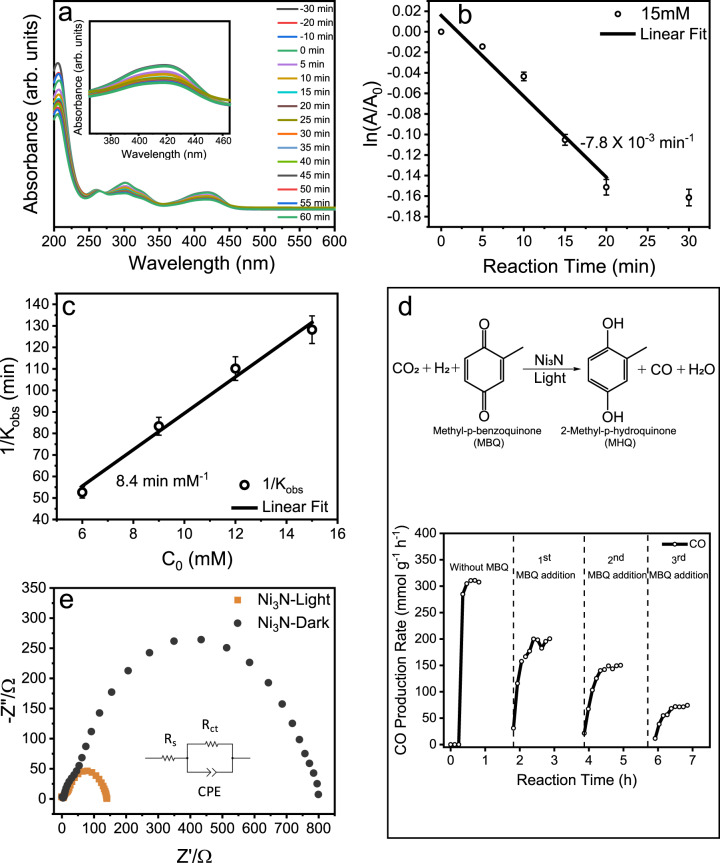
Electron transfer studies of Ni_3_N nanosheets. **a** UV-Vis spectra showing the conversion of Fe^3+^ to Fe^2+^ as a function of irradiation time, using 15 mM K_3_[Fe(CN)_6_]. Inset: Magnified UV-Vis spectra around 419 nm; **b** Pseudo-first-order plot of ln(A/A_0_) against reaction time; **c** Langmuir–Hinshelwood plot of the reciprocal of observed pseudo-first-order rate constant as a function of initial ferricyanide concentration. **d** CO production rate of photocatalytic CO_2_ hydrogenation reaction in the presence of methyl-p-benzoquinone (MBQ). Each MBQ addition corresponds to the addition of 50 µL of 1 M solution of MBQ in diethyl ether; **e** Nyquist plots of Ni_3_N nanosheets in dark and light, where Z′ is real impedance, and Z″ is imaginary impedance. Error bars: calculated from data of at least three repeated experiments.

To study the electron transfer ability of Ni_3_N nanosheets, the reduction of ferricyanide [Fe(CN)_6_]^3−^ to ferrocyanide [Fe(CN)_6_]^4−^ was performed as a model reaction^31^. Under light irradiation, the absorbance at 419 nm due to Fe^3+^ decreases and absorbance at 240 nm due to Fe^2+^ increases, indicating the reduction of Fe^3+^ to Fe^2+^ (Fig. 7a), indicative of electron transfer from Ni_3_N to the Fe^3+^ of the iron complex. We modeled the reaction with pseudo-first-order kinetics to get the rate of reaction (Fig. 7b), which was 7.8 × 10^−3 ^min^−1^. The reduction of Fe^3+^ is a surface reaction on the surface of Ni_3_N nanosheets. This process can be broken down into three steps; (1) adsorption of Fe^3+^ on Ni_3_N, (2) electron transfer from Ni_3_N to Fe^3+^ (to reduce it to Fe^2+^), and (3) desorption of Fe^2+^ from Ni_3_N surface.

The reaction rate that we obtained from the pseudo-first-order kinetics was the overall rate of all these three steps. In order to extract the net electron transfer rate from the observed rate, we employed the Langmuir–Hinshelwood type rate law (Eqs. 1, 2), as it describes the surface catalyzed reactions, considering the adsorption-desorption processes.

1 $${r}_{0}={k}_{{obs}}{C}_{0}={k}_{r}\frac{{K}_{{LH}}{C}_{0}}{{1+K}_{{LH}}{C}_{0}}$$

2 $$\frac{1}{{k}_{{obs}}}=\frac{1}{{K}_{{LH}}{k}_{r}}+\frac{{C}_{0}}{{k}_{r}}$$

where *k*_r_ is the photocatalytic reaction rate, *K*_LH_ is the apparent adsorption constant of the reactant molecule on the Ni_3_N surface, *C*_0_ is the initial concentration of the reactant Fe^3+^, and *k*_obs_ is the observed pseudo-first-order rate constant.

The net electron transfer rate was obtained from the plot of 1/*k*_obs_ versus the initial concentration of Fe^3+^. The one-electron reduction reaction was carried out with various concentrations of the ferricyanide salt (Supplementary Figs. S23–S25). The reciprocal of the observed rate versus the initial concentration of the reactant (Fig. 7c) was then plotted to get the electron transfer rate, which was found to be 11.9 × 10^−2 ^mM min^−1^.

To further investigate the role of hot electrons, we performed CO_2_ hydrogenation in the presence of an electron-accepting molecule, methyl-p-benzoquinone (MBQ)^32^. We observed that with the addition of MBQ (50 µL of 1 M solution in diethyl ether), the CO production rate decreases (Fig. 7d). With the further addition of MBQ, the CO production rate further decreases in each cycle to just 50 mmol g^−1^ h^−1^. This indicated that MBQ molecules compete with CO_2_ molecules for hot electrons, and CO_2_ gets less electrons while MBQ molecules (which are easy to reduce) get more electrons, reducing them to methyl-p-hydroquinone, which in turn resulted in a decrease in CO_2_ hydrogenation reaction rate. This observation further indicated the role of hot electrons in this plasmonic process.

Photo-electrochemical studies were carried out to understand the excited charge carrier transfer in Ni_3_N nanosheets. The Nyquist plots (Fig. 7e) elucidate that the charge-transfer resistance of the Ni_3_N nanosheets decreases significantly under light excitation compared to the dark condition^17^. This result suggests that the Ni_3_N nanosheets have a faster charge transfer rate in light than in the dark. This was due to the plasmonic excitation of the Ni_3_N nanosheets in light, thereby generating a high number of hot charge carriers on the surface, which increases the charge density on the surface of the material. This high charge density results in a faster charge transfer rate and low charge transfer resistance^17^.

### Thermal stability of Ni_3_N nanosheets

**Fig. 8:**
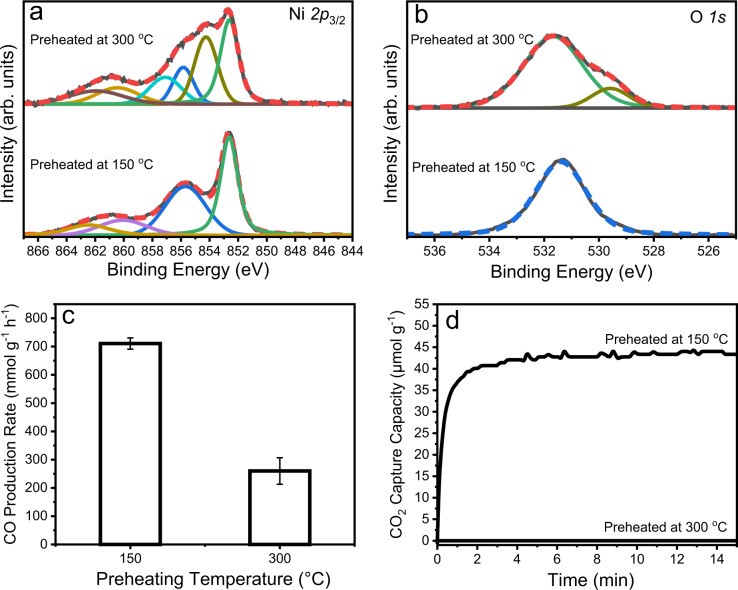
Role of hydroxide terminations of Ni_3_N nanosheets. XPS spectra of preheated Ni_3_N nanosheets **a** Ni*2p*_3/2_, **b** O*1s;* **c** Photocatalytic CO_2_ hydrogenation and **d** CO_2_ capture capacity at 120 °C, using preheated Ni_3_N nanosheets; H_2_ (4 mL min^−1^), CO_2_ (73 mL min^−1^), xenon lamp (400–1100 nm) at 2.7 W cm^−2^ light intensity. Error bars: calculated from data of three repeated experiments.

To study the role of surface nickel hydroxide terminations of Ni_3_N nanosheets on the CO production rate, the nanosheets were preheated at 300 °C under argon to transform the nickel hydroxide to nickel oxide (based on TPReaction and TGA data, Supplementary Fig. S9) and compared with as-synthesized sample (150 °C preheated, keeping hydroxide termination intact). Nickel hydroxide to nickel oxide transformation was confirmed by XPS analysis (Fig. 8a, b). An increase in Ni*2p*_3/2_ peaks intensities between 852 to 854 eV at 300 °C indicated nickel hydroxide converted to nickel oxide, although partially (Fig. 8a). The O*1s* peak around 529.6 eV in the 300 °C preheated sample further confirmed this partial conversion (Fig. 8b). When these preheated Ni_3_N samples were evaluated for plasmonic CO_2_ hydrogenation, the catalytic activity decreased after the heat treatment at 300 °C (Fig. 8c). We attributed this decrease in the activity to their CO_2_ capture capacity and the change in the excited electron dynamics (discussed in the next nanosecond transient absorption spectroscopy section). The CO_2_ capture capacity was drastically reduced after heating for 300 °C preheated sample, as the surface nickel hydroxide known to capture CO_2_ was converted to nickel oxide (Fig. 8d). Hence, its photocatalytic activity was also reduced (Fig. 8c).

### Photophysics and electron transfer dynamics of Ni_3_N nanosheets

**Fig. 9:**
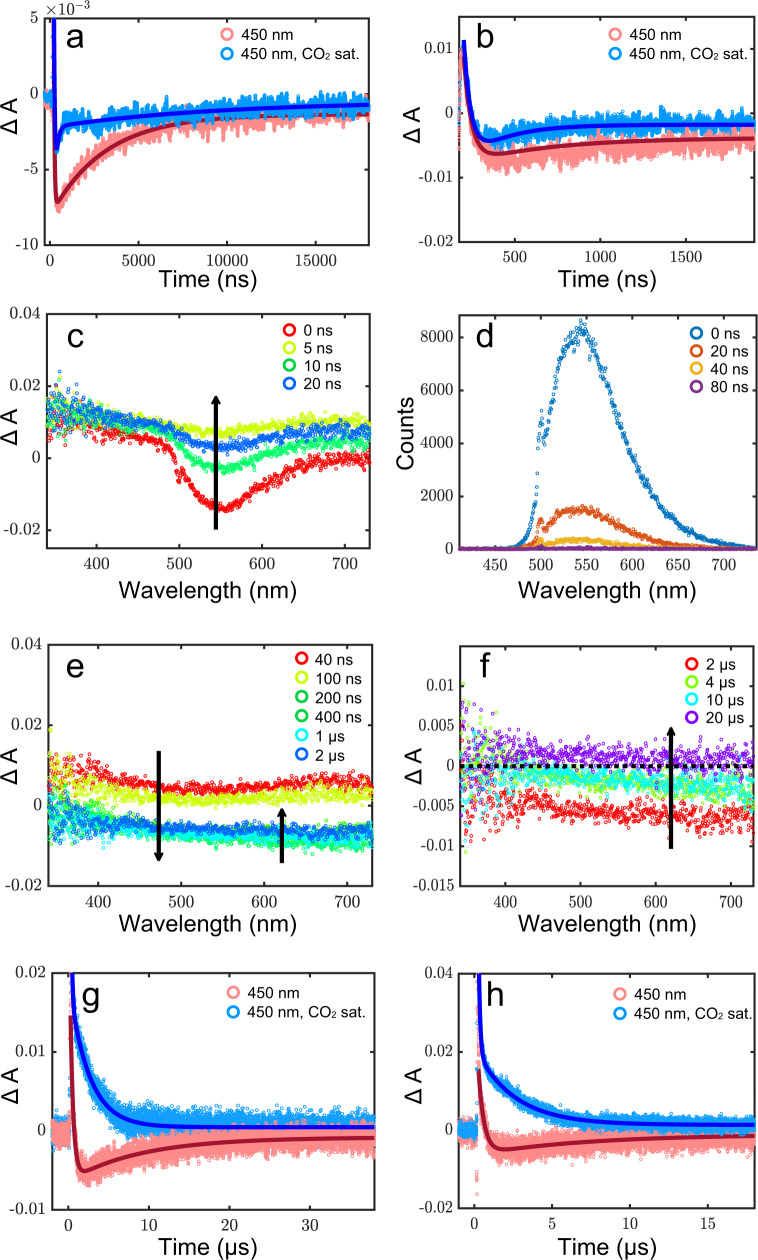
Nanosecond transient absorption spectroscopy study. **a**, **b** Representative kinetics monitored at 450 nm for the as-prepared Ni_3_N nanosheets after photoexcitation at 500 nm (pulse energy = 10 mJ/pulse), with (blue) and without (pink) CO_2_ saturation (Solid lines are fits); Transient absorption and emission spectra monitored **c**, **d** at indicated initial timescales and **e**, **f** at later timescales, upon excitation of the as-synthesized Ni_3_N nanosheets in ethanol suspension at 500 nm (pulse energy = 10 mJ/pulse); **g**, **h** Representative kinetics monitored at 450 nm for the heated Ni_3_N nanosheets after photoexcitation at 500 nm (pulse energy = 10 mJ/pulse), with (blue) and without (pink) CO_2_ saturation (solid lines are fits). The right panels (**b**, **h**) show data of the initial timescales on the left panel (**a**, **g**), respectively, with a greater resolution so that the rise can be seen.

Nanosecond transient absorption experiments were carried out on the as-synthesized (150 °C preheated) and 300 °C heated samples of Ni_3_N in ethanol suspensions to glean direct insight into the material photophysics and electron transfer dynamics. The measured absorption spectrum (Supplementary Fig. S26), featuring a broad panchromatic absorption, agrees well with that of the solid state, supporting the idea that similar behavior can be expected in both conditions. Photoexcitation of the as-synthesized Ni_3_N sample at 500 nm reveals the presence of long-lived electrons with a lifetime of around one microsecond that is readily quenched to ca. 250 ns upon saturating the solution with CO_2_ (Fig. 9a, b, pink and blue traces, respectively, with the fits indicated as solid lines), providing evidence of the proposed electron transfer process. It is worth noting that the dynamics do not change with the wavelength monitored (Supplementary Fig. S27). All traces show a distinctive negative signal, followed by a rise on the timescale of approximately 50 ns, which is succeeded by a long-lived decay of a microsecond. It is the latter that is quenched upon CO_2_ saturation.

Spectral evidence helps delineate the processes in question: the initial signal is dominated by radiative decay of the electrons (Fig. 9c–f) together with a ground-state bleach, as evidenced by the loss of the stimulated emission centered at 550 nm (confirmed with independent time-resolved emission measurements, Fig. 9c–f). The following rise on the timescale of around 50 ns can be attributed to hole-quenching by the surrounding ethanol (whose role can be viewed in analogy to hydrogen in the heterogeneous gas-phase system) and is concomitant with the loss of the positive transient signal. The residual bleach can thus be assigned to the long-lived electrons, which are involved in the hydrogenation of CO_2_. The aforementioned positive transient absorption signal ascribed to the holes is notable and likely stems from a low optical band gap in this material, resulting in a broad, continuous absorption.

Qualitatively similar behavior is observed for the heated Ni_3_N sample but with notable quantitative differences: the rise and decay times increase to around 300 ns and 10 µs, respectively. While the increased electron lifetime may naively suggest more activity, both the spectra (Supplementary Fig. S28) and kinetics (Fig. 9g, h) is offset by a much larger positive transient at the outset instead of pointing to the presence of a more significant proportion of trap states as compared to the as-synthesized sample. Viewed cohesively, the data indicates that while the electrons in the heated sample have a lifetime that is an order of magnitude longer than the as-prepared sample, a significant proportion of them may get localized on trap states, rendering them inactive for the redox process.

Photophysics reveals that the hydroxyl groups decrease the number of electrons in inactive trap states, thus increasing the number of hot electrons available for photocatalysis. This confers a dual function to the hydroxyl groups, namely: (1) primary CO_2_ adsorption site and (2) electron trap suppression.

### CO_2_ hydrogenation molecular mechanism using in situ DRIFTS

**Fig. 10:**
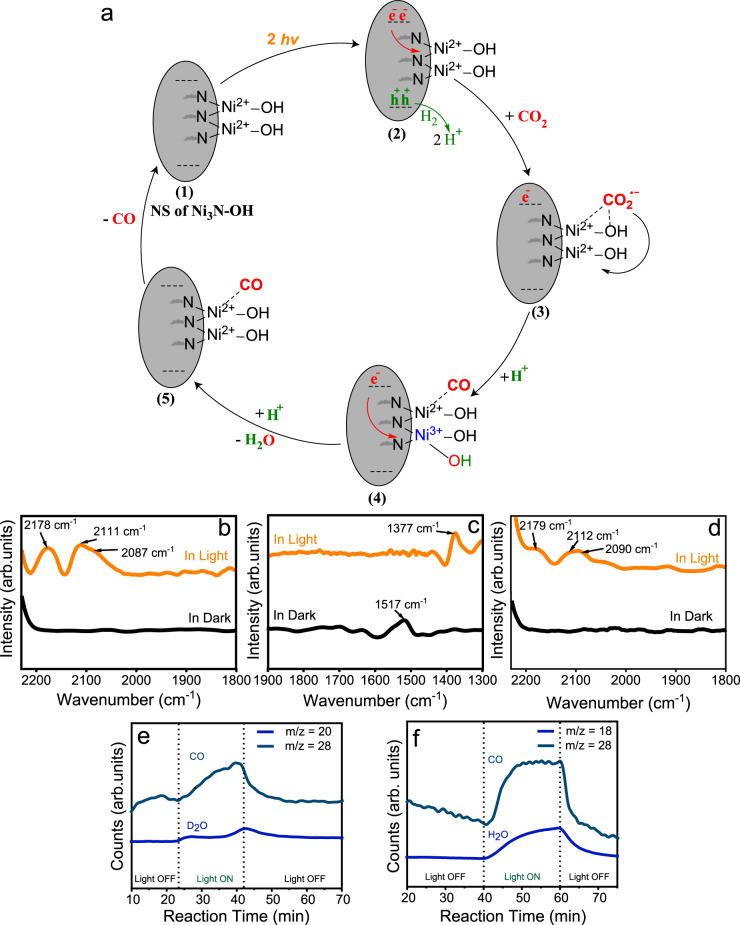
Proposed mechanism of plasmonic CO_2_ hydrogenation using Ni_3_N nanosheets. **a** Schematic of molecular mechanism; In situ DRIFT spectra with peaks for **b** gaseous CO and adsorbed CO, and **c** adsorbed carbonate species during CO_2_ hydrogenation by H_2_; **d** In situ DRIFT spectra of gaseous CO and adsorbed CO with only CO_2_ gas feed; Ions signal in mass spectroscopy of different products during photocatalytic CO_2_ hydrogenation using **e** D_2_ and **f** H_2_.

The excellent photocatalytic activity of Ni_3_N is due to the synergy of Ni^1+^ and Ni^2+^ sites present on the nanosheets. Based on catalysis results and transient spectroscopy study, we propose the mechanism shown in Fig. 10a. The light is absorbed causing plasmonic excitation in Ni_3_N, which then generates excited electrons and holes in the nanosheets. The hydroxide sites on the surface interact with CO_2_ molecules and help in their capture (chemisorption) on the surface. The excited electron is then transferred to the CO_2_ molecule via the hydroxide layer generating  CO_2_^•‒^ radical anion. The electron transfer from Ni_3_N to CO_2_ was observed in a nanosecond transient absorption spectroscopy study (Fig. 9). Meanwhile, the holes are taken care of by the H_2_ molecule generating H^+^. The  CO_2_^•‒^ radical anion then undergoes direct dissociation into CO and O^−^. The O^−^ is then converted into OH by H^+^ followed by the removal of the water molecule after reacting with another H^+^. Then, in the last step of the reaction, the CO molecule finally desorbs from the surface, leaving the catalyst in its initial state (Fig. 10a).

The photocatalytic CO_2_ hydrogenation mechanism using Ni_3_N was investigated by an in situ Diffuse Reflectance Infrared Fourier Transform Spectroscopy (DRIFTS) (Fig. 10b–d). The spectra were recorded in H_2_/CO_2_ flow under light irradiation and in the dark. The peaks corresponding to monodentate carbonate were observed at 1377 cm^−1^ in light and 1517 cm^−1^ in the dark (Fig. 10c)^33–35^. A strong signal centered at 2087 cm^−1^ assigned to C=O stretching vibrations of linearly bonded CO atop a single Ni^2+^ site (Ni^2+^-CO) (Fig. 10b)^33–35^. On flowing only CO_2_, we also get the same peak at 2090 cm^−1^ (Fig. 10d), which confirmed that the CO_2_ hydrogenation was taking place by a direct dissociation pathway^36^. DRIFT spectra showed intense peaks for gaseous CO at 2178 and 2111 cm^−1^, and none of these peaks were present when recorded in the dark at 184 °C (Fig. 10b–d, bottom spectra). The H_2_ molecules as a hole quencher were studied by replacing H_2_ with its isotope D_2_ during the CO_2_ hydrogenation reaction and monitoring the reaction products with mass spectroscopy. When H_2_ was used, we detected the ion signal for H_2_O in MS, but when D_2_ was used, only D_2_O signals were detected (Fig. 10e, f). This indicated that H_2_ was acting as a hole quencher.

#### Thermal vs. non-thermal debate in plasmonic catalysis

Light excitation in plasmonic nanoparticles causes the generation of non-equilibrium electron-hole pairs with high energies after the decay of coherent oscillation of electrons. In solid-state physics, such non-thermal, highly energetic charge carriers are referred to as hot carriers because they deviate significantly from the thermalized Fermi–Dirac energy distribution of the metal’s free electrons^5,37–39^. The transfer of these hot charge carriers from the nanoparticle to nearby molecular adsorbates has the potential to drive electronic and chemical processes on the nanoparticle surface. The thermalization of these hot electrons via electron–phonon scattering results in the heating of the nanoparticle and further heat diffusion into the surrounding reaction medium, which is termed as the photothermal effect of the plasmonic excitation. Plasmonic nanoparticles were shown to facilitate various chemical transformations on their surface under light illumination. However, significant challenges were encountered when researchers began to investigate the mechanism responsible for plasmon-assisted chemical transformations. The main debate on this problem is between two schools of thoughts: one believes that the transfer of hot charge carriers from the nanoparticles to the adsorbed molecule is responsible for the catalysis known as the “non-thermal” pathway. In contrast, the other believes that the local temperature of the nanoparticle is the primary driving force for the catalysis, known as the “thermal” pathway.

The main challenge for the “non-thermal” pathway is the short lifetime of primary hot carriers^37^. They thermalize via electron–electron scattering within a few tens of femtoseconds (fs), making any interaction with the surrounding environment unlikely. The time-average number of primary hot electrons generated in a single nanoparticle under illumination can be calculated using the following equation:

3 $$\langle {N}_{hot{e}^{-}}\rangle=\frac{{\sigma }_{abs}\times I\times {\tau }_{e-e}}{h\nu }$$

where σ_abs_ is the absorption cross-section of the nanoparticle, *I* is the light intensity, *τ*_e-e_ is the electron–electron scattering lifetime, and *hν* is the photon energy. Under continuous wave (CW) illumination, there is a high possibility of thermalization of a hot carrier before the next photon adsorption, resulting in a smaller number of excited carriers available for chemical transformation. Under pulse excitation, the number of hot carriers can increase, and some of them may remain available to participate in chemical reactions^37–39^; however, photocatalysis is generally carried out under CW illumination conditions. Thus, a hot carrier-mediated “non-thermal” pathway seems ideally not feasible. However, in a small nanoparticle, there is the possibility of an increased lifetime of the excited electrons due to increased confinement, the higher granular density of states, and decreased electron–electron interactions^39^. Additionally, the equilibration time with the lattice is longer due to decreased electron–phonon coupling. This increased hot carrier lifetime makes their transfer to reactant molecules possible, creating negative-ion states of adsorbed molecules, which can then undergo subsequent chemical transformations via this “non-thermal” pathway. On the other hand, the thermal pathway suffers from difficulties in the accurate spatial and temporal measurement of the associated local temperature of the plasmonic nanoparticle. Hence, based on experimental results, there are reports of thermal^40–44^, as well as non-thermal pathways^7,12,31,45–58^.

The work by various groups, like Halas^49–53^, Linic^12,54^, Jain^7,31,55–57^, Nordlander^39^, Chandra^45^, Camargo^46^, Cortes^48^, and others, observed the non-thermal pathways during their plasmonic catalysis. Halas et al. reported one such early finding, where it was demonstrated that H_2_ could be dissociated on Au nanoparticles under light excitation without the need for external heating^53^. The involvement of hot electrons in the catalysis was established by doing experiments without light, wavelength-dependent catalysis, light intensity-dependent catalysis, and density functional theory calculations. Many other groups have reported hot electron-mediated catalysis, such as the reduction of ferric (Fe^3+^) ions by Au NPs, the reduction of CO_2_ by Au NPs, water splitting, propylene epoxidation, dry reforming, oxygen dissociation, and so on^7,12,31,37–39,45–58^. These reports demonstrated the involvement of hot electrons through a range of studies such as dark reactions at various temperatures, light intensity-dependent catalysis, wavelength-dependent catalysis, kinetic isotope effect, extracting thermal and non-thermal contributions from activation energy calculations, finite-difference time domain, ultrafast transient spectroscopy, etc. They deciphered the role of the non-thermal pathway and found it as an activation mechanism in plasmonic catalysis.

However, there was one major concern regarding all these reports, and that was the accurate estimation of the local surface temperature. Sivan et al. and Dubi et al. studied the role of the thermal pathway in plasmonic catalysis^41–44^. The main argument in this “thermal” school of thought is that the actual local temperature near the nanoparticle can be very high and that it is generally underestimated when measured by an infrared camera or a thermocouple touching the surface of the catalyst, which are the most common methods of measuring the temperature of the catalyst under light excitation. A report by Sivan et al. suggested a numerical model to estimate the local temperature of the nanoparticle under light excitation using coupled Boltzmann-heat equations based on energy conservation and basic thermodynamics^43^. Using these equations, they calculated the local temperature of the nanoparticle, and from that temperature (which is generally higher than that of an IR camera or thermocouple), activation energy and reaction rates were calculated. Sivan et al. reported that most of the absorbed light photons, according to their theoretical model, resulted in a change in electron distribution near the Fermi energy (and not the generation of high-energy carriers), but rather the heating of the nanoparticles^43^. As a result, according to these authors, the likelihood of producing high-energy electrons and using them to carry out chemical transformations is low. Hence, the thermal pathway, according to them, is the primary driving force in plasmonic catalysis.

The debate between these two plausible mechanisms is still ongoing, and both sides have presented their arguments in numerous reports with more detailed experiments, calculations, models, and results. Some examples of such discussions are given below:

Halas et al. reported a Cu-Ru supported on MgO photocatalyst for the production of H_2_ from NH_3_^50^. They observed a decrease in the activation energy barrier under light excitation, demonstrating that the contribution of “hot” electrons to the reaction was significantly greater than the contribution of purely thermal effects. However, Sivan et al. expressed concern about this report and their data interpretation^59^. Questions were raised about the measurement of surface temperature by IR camera (due to overestimated emissivity value) and the use of intensity and wavelength-dependent activation energy. Sivan et al. showed that reaction rates still obey an Arrhenius form with an intensity and wavelength-independent activation energy, and the effective reactor temperature grows linearly with light intensity, indicating that a major contribution comes from the thermal pathway. Halas et al.^60^ responded to Sivan et al.’s^59^ concerns in their response. They justified their emissivity value used for the IR camera (which was also calibrated) during temperature measurement by citing related literature on nanoparticle emissivity. They also showed that Sivan’s model of liner increase in temperature of the catalyst with light intensity is true only for very small temperature increases (100 K) and not for the temperatures observed in Halas et al.’s^50^ original work. Also, Sivan’s use of light-independent activation energy is not physical because hot carriers can change adsorbate coverage on the catalyst surface and thus change the apparent activation barrier^60^.

Another point Halas et al.^60^ brought out was based on the stability of the catalyst at the temperatures predicted by Sivan et al.^59^. Sivan’s model predicted a temperature as high as 1150 K, and at this elevated temperature, the metal nanoparticles ideally should melt, but this was not observed by Halas et al. This indicated an overestimation of the local temperature by the method reported by Sivan et al. A similar line of questions on the light-dependent activation energy, extraction of thermal rates of reaction, and temperature measurement was raised by Sivan et al.^61^ in Halas et al. recent work about plasmonic hydro-defluorination reaction^52^. Halas et al.^62^ replied that Sivan et al.^61^ misunderstood how the reaction rate was calculated, and their interpretation of the *R*_dark_ ≈ *R*_thermo_ is not right, which led to incorrect data analysis. Photothermal simulations were also carried out, which support the accuracy of the thermal camera for surface temperature measurements of the catalyst^52^.

Sivan et al.^63^ published an article where they carried out data analysis of some of the recent articles on plasmonic catalysis. In this article, the authors used a thermal-based Arrhenius equation to show that all the data presented in the original reports can be interpreted as the thermal pathway. The key assumption of Sivan et al. in this report was an underestimation of local temperature because the presence of a large number of nanoparticles in the catalysis experiments results in a collective macroscopic heating effect that is orders of magnitude greater than the minor heating provided by a single NP. In this report, Sivan et al. raised concerns about the emissivity values used, as an overestimation of emissivity value will introduce huge errors in the temperature measurements by IR camera^63^. Sivan also pointed out that the presence of temperature gradients within the catalyst due to the non-uniform illumination of the catalyst can also introduce errors in the temperature measurement if a thermometer was placed away from the catalyst surface^63^. Sivan et al. used the shifted Arrhenius equation (Eq. 4) to study the distribution of heat under light illumination in these plasmonic systems^40,63^. This equation is corrected for illumination-induced heating.

4 $$R \sim {{\exp }}(-\frac{{\varepsilon }_{a}}{{k}_{B}T\left(r\right)+a{I}_{{inc}}})$$

However, Jain^64^ studied the applicability of the above equation in plasmonic catalysis. He started with an assumption that a decrease in activation energy (*E*_a_) is linearly dependent on the light intensity in photocatalysis (Eq. 5):

5 $${E}_{a}^{{light}}={E}_{a}^{{dark}}-{BI}$$

here, *B* is a proportionality constant with units of eV cm^2^ W^−1^ if *E*_a_ is expressed in units of eV and *I* in units of W cm^−2^. On further solving the above equation and using the Arrhenius equation, he obtained Eq. 6.

6 $$R={R}_{0}{{\exp }}[-\frac{{E}_{a}^{{dark}}}{{k}_{B}{T}_{s}\left(1+{bI}\right)}]$$

where *b* is$\frac{B}{{E}_{a}^{{dark}}}$ and has units of cm^2^ W^−1^.

When this equation (Eq. 6) was compared to the general Arrhenius equation, the reaction appeared to take place at a theoretical temperature that was proportional to the light intensity higher than the actual temperature *T*_s_, which was referred to as dummy temperature by Jain (Eq. 7)^64^.

7 $${T}_{{dummy}}={T}_{s}(1+{bI})$$

This equation (Eq. 6) has an identical form which was used by Sivan et al.^59^ in their report to support the thermal pathway over the non-thermal pathway. This led to the conclusion that the plasmonic excitation was only increasing the temperature but not causing any change in the activation energy barrier. Thus, such a treatment of the Arrhenius equation inherently masked the non-thermal effects of light excitation by the temperature increase and only assumed a thermal pathway.

In this case of plasmonic Ni_3_N nanosheets catalyzed CO_2_ reduction, while we cannot have hot carriers without some heat liberation, our various studies (discussed in previous sections) indicated the involvement of the hot carries in the catalytic process and that the thermal contribution while present, it cannot alone drive the process. Furthermore, the Ni_3_N nanosheet’s thermal instability also indicated that there was another mechanism at play. We found that Ni_3_N nanosheets start degrading from 325 °C; hence if Ni_3_N nanosheets’ local surface temperature increases above this temperature during plasmonic catalysis due to local plasmonic heating, nanosheets will degrade and become catalytically inactive. However, Ni_3_N nanosheets were stable for 25 h (with a constant CO production rate), indicating that surface temperature must be below 325 °C during the plasmonic catalysis. When the CO_2_ hydrogenation was carried out at 400 °C using external heating, the CO production rate of only 80 mmol g^−1^ h^−1^ was observed, indicating degradation of nanosheets during catalysis. This observation indicated the role of non-thermal plasmonic excitation of Ni_3_N nanosheets for CO_2_ hydrogenation, apart from some thermal contribution.

In conclusion, we have demonstrated that plasmonic Ni_3_N nanosheets catalyze photocatalytic CO_2_ hydrogenation using visible light. The reaction was carried out at low temperatures under light irradiation of different intensities without any external heating. The Ni_3_N nanosheets showed an excellent CO production rate of 1212 mmol g^−1^ h^−1^ and a selectivity of 99% in the flow conditions. The catalyst was stable for up to 25 h.

The sheet morphology of Ni_3_N with variable sheet thickness resulted in efficient absorption of broadband light followed by the generation of excited electrons. The surface hydroxide layer helped in the CO_2_ capture and efficient electron transfer resulting in good photocatalytic activity of Ni_3_N nanosheets. Transient absorption measurements not only allowed for direct observation of the electron transfer process from the nanosheets to CO_2_ but also revealed the favorable role of the hydroxyl groups as trap suppressors, allowing for a greater proportion of the generated hot electrons to be accessible for harvesting.

CO_2_ hydrogenation reaction rates using Ni_3_N nanosheets showed super-linear power law dependence on the light intensity, with a power law exponent value of 6.3. Further, photocatalytic quantum efficiencies of this process using Ni_3_N nanosheets increased with an increase in light intensity and reaction temperature. These two relationships indicated the role of non-thermal pathways in this plasmonic Ni_3_N nanosheets catalyzed CO_2_ hydrogenation reaction. Notably, in the presence of an electron-accepting molecule, methyl-p-benzoquinone (MBQ), the CO production rate decreases significantly. This was due to the fact that MBQ molecules competed with CO_2_ molecules for hot electrons, and CO_2_ got fewer electrons while MBQ molecules got more electrons, reducing it to methyl-p-hydroquinone, which in turn resulted in a decrease in CO_2_ hydrogenation reaction rate. Ni_3_N nanosheets (which were thermally stable only up to 325 °C) showed stable catalytic activity for a long time (25 h), indicating surface temperature must be below 325 °C during the plasmonic catalysis, and below this temperature, it showed poor catalytic activity in the dark. The successive light on and off-cycle experiment also indicated the role of the non-thermal reaction pathway. The one-electron photoreduction of Fe^3+^ to Fe^2+^ and electrochemical impedance measurement in the dark and light indicated the electron transfer ability of Ni_3_N nanosheet under light irradiation. Thus, although we cannot completely discard the thermal contribution of hot spots during catalysis, in general, the results indicated the direct involvement of hot electrons and holes.

In situ DRIFTS study showed C=O stretching of linearly bonded CO on the Ni^2+^ site, indicating the role of surface hydroxide. CO_2_ hydrogenation took place by direct dissociation path via linearly bonded CO. Thus, the excellent catalytic performance of Ni_3_N nanosheets suggested that next-generation plasmonic catalysts can be developed using metal nitrides over conventional metal nanoparticles.

## Methods

### Materials

Nickel(II) bis(acetylacetonate) (Ni(acac)_2_, 95%), o-xylene (anhydrous, 97%), ethylenediamine (≥ 99%), Lithium nitride (Li_3_N, ≥ 99.5%), Potassium ferricyanide(III) (K_3_Fe(CN)_6_, 100%), 5 wt% Nafion solutions, Methylene blue (MB) and Methyl-p-benzoquinone (MBQ) were purchased from Sigma-Aldrich Chemicals Pvt. Ltd. Ethanol was purchased from Scitech, Mumbai. Diethyl ether was purchased from Merck Life Science Pvt. Ltd. Deionized water was supplied from a Millipore-Q system.

### Synthesis of nickel nitride nanosheets

In the synthesis of nickel nitride (Ni_3_N) nanosheets, nickel(II) bis(acetylacetonate) (257 mg, 1 mmol) was added into a 100 mL round bottom (RB) flask. Anhydrous o-xylene (40 mL) was added to the round bottom flask using a 50 mL glass syringe under argon flow. The mixture was stirred at room temperature for 2 min. Ethylenediamine (5 mL) was then added to the stirring mixture, and the mixture was again stirred for 2 min, followed by the addition of Li_3_N (35 mg, 1 mmol) dispersed in 1 mL o-xylene under argon flow. The RB was sealed, and the mixture was stirred at 25 °C for another 2 h. Afterward, the resulting mixture was transferred into two 40-mL Teflon-lined autoclaves, and the autoclaves were sealed under argon. The mixture was then heated to 270 °C at a rate of 3 °C min^−1^ for 20 h. After the reaction was completed, the black powder was separated and collected by centrifugation (10 min, 15356×*g*). Finally, the powder was washed with ethanol (two times) and distilled water (two times) and dried under a vacuum at 45 °C. The synthesized Ni_3_N nanosheets were stored under atmospheric conditions.

### Catalysts characterizations

All SEM imaging was performed using a Zeiss Ultra microscope at 3 kV with a working distance of 3 mm. Scanning transmission electron microscopy (STEM) analysis was carried out using FEI Tecnai operated at an accelerating voltage of 200 kV. Elemental mapping was carried out using energy-dispersive X-ray spectroscopy (EDS). Samples were prepared by dispersing a small amount of solid powder in ethanol by sonicating for 10 s. The dispersion was drop-casted onto a holey carbon-coated 200 mesh copper TEM grid. PXRD patterns were obtained using a Panalytical X’Pert Pro powder X-ray diffractometer with Cu-Kα radiation. N_2_ sorption measurements were performed using a Micromeritics 3-Flex surface analyzer (samples were degassed at 110 °C overnight under a vacuum before analysis). UV-DRS measurements were carried out using a JASCO UV-Vis/NIR spectrophotometer. XPS analysis was carried out using a Thermo Kα+ spectrometer with micro-focused and monochromated Al-Kα radiation (1486.6 eV) as the X-ray source. The sample was prepared by sprinkling solid powder on carbon tape. The carbon signal at 284.6 eV was used as an internal reference. The EPR spectra of all samples were recorded at 110 K using a JEOL EPR spectrometer. The following conditions were used to record the EPR spectra of the catalyst: amount = 20 mg, power = 5 mW, modulation frequency = 100 kHz, and the number of scans = 5. The Temperature programmed reaction (TPReaction) was done using Catalyst Analyzer BELCAT II coupled with a Quadrupole mass spectrometer (Belmass).

### Temperature programmed reaction (TPReaction)

The temperature programmed reaction (TPReaction) was carried out using 47 mg of as-prepared Ni_3_N in a quartz tube. The catalyst was pretreated in the presence of argon (50 sccm) at 110 °C (10 °C/min) for 60 min inside the furnace. After pretreatment, the temperature was increased to 1000 °C (5 °C/min) in argon flow (30 sccm). The products were monitored by a thermal conductivity detector (TCD) as well as Mass Spectrometer.

### CO_2_ hydrogenation using nickel nitride nanosheets

Photocatalytic CO_2_ hydrogenation was carried out in a flow reactor with a quartz window, a heater, and a thermocouple to accurately measure the temperature of the catalyst bed connected to a temperature controller. The gases were introduced in the reaction chamber by mass flow controllers (MFCs), and the outlet was connected to an Agilent 490 MicroGC having a 20 m Molesieve 5 A column and a thermal conductivity detector (TCD). The catalyst (~2.0 mg) was taken in a ceramic porous base crucible, which was placed inside the reactor chamber. Argon (Ar) gas (100 mL min^−1^) flowed through the reactor for 10 min, and then the reactor was heated up to 150 °C (20 °C min^−1^) for 30 min to remove any moisture adsorbed on the catalyst surface. The reactor was cooled to 25 °C under Ar flow (100 mL min^−1^). The reactant gases were introduced into the reactor chamber through Alicat mass flow controllers; CO_2_ at 73 mL min^−1^, H_2_ at 4 mL min^−1^, with a total flow of 77 mL min^−1^ at 1 bar pressure. The catalyst was then irradiated with light (300 W Xenon Lamp ~2.7 W cm^−2^, 400–1100 nm), and the progress of the reaction was monitored by using an online MicroGC every 6 min.

Flow study experiments were performed by changing the total flow of the reactant gases (22 mL/min, 44 mL/min, 77 mL/min, 99 mL/min, 110 mL/min, 120 mL/min, 130 mL/min, 150 mL/min, and 220 mL/min) keeping the ratio of CO_2_ to H_2_ fixed at 10:1. Ratio study experiments were performed by changing the ratio of CO_2_ to H_2_ (CO_2_: H_2_ = 1:1, 6:1, 10:1, 20:1, and 37:1), keeping the total flow fixed at 77 mL/min. Further studies were done at a total flow of 77 mL/min and a ratio of CO_2_: H_2_ = 20:1. Higher temperature studies (200 °C, 300 °C, and 400 °C in light and 200 °C, 300 °C, and 400 °C in the dark) experiments were performed by providing external heating to the catalyst bed by the heater inside the reaction chamber. CO_2_ hydrogenation of samples preheated at five different temperatures (150, 300, 400, 500, and 600 °C) under Ar (100 mL/min) was carried out. For tests under different light intensities, the light power was tuned by changing the light intensity of the Xenon lamp. For testing under different wavelengths, different laser (Thorlabs Diode Laser) diodes emitting monochromatic light at different wavelengths were used. The ^13^C isotopic labeling experiments were also conducted under identical conditions in the same reactor, with the ^12^CO_2_ gas being replaced by ^13^CO_2_, and the products were detected using Agilent 7890B GC-MS equipped with a CP7430 column. For quantification, the GC was calibrated by injecting known concentrations of standard gases like CH_4_, CO, H_2_, alkanes, etc. The slope of peak area versus ppm plot gives the calibration constant (area/ppm), which was used to calculate the product formation of the product formed.

$${{{{{\rm{Production}}}}}}\; {{{{{\rm{rate}}}}}}\; {{{{{\rm{of}}}}}}\; {{{{{\rm{CO}}}}}}({{{{{\rm{mmol}}}}}}{{{{{{\rm{g}}}}}}}^{-1}\,{{{{{{\rm{h}}}}}}}^{-1})=\frac{{{ppm}}_{{CO}}\times {Total}\,{flow}\left({mL}\,{\min }^{-1}\right)\times 60}{22400\times {weight}\,{of}\,{the}\,{catalyst}\,{in}\,{gm}\times 1000}$$

The apparent activation energy (E_app_) was calculated by using the Arrhenius equation,${{{{{\rm{ln}}}}}}\left({k}_{{CO}}\right)={{{{{\rm{ln}}}}}}\left(A\right)-\frac{{E}_{{app}}}{{RT}}$, where *k*_*CO*_ is CO production rate. The slope of ln(k) versus 1000/T plot gives us the value of $\frac{{E}_{{app}}}{1000\times R}$; then, ${E}_{{app}}={Slope}\times R\times 1000$ J mol^−1^.

### Fe^3+^ to Fe^2+^ one-electron reduction reaction

First, 5 mL of dispersion of Ni_3_N in water (0.2 mg mL^−1^) was mixed with 1 mL of K_3_[Fe(CN)_6_] solution (6, 9, 12, or 15 mM in water) in a glass reactor (of 18 mL volume) and the reactor was then sealed with rubber septa. Argon gas (100 mL min^−1^) was bubbled through the reaction mixture for 15 min to remove dissolved oxygen. The solution was then stirred at 500 rpm, and dark adsorption kinetics of Fe^3+^ on Ni_3_N was studied by sampling the reaction mixture (200 µL) from the reactor every 10 min for 30 min. The reactor was then exposed to Xenon light (0.41 W cm^−2^). A liquid sample (200 µL) of the reaction mixture was taken in an Eppendorf after every 5 min and diluted with DI water (1 mL). This mixture was then centrifuged for 1 min to separate the solid catalyst, and the separated liquid sample was then taken in a cuvette. The UV-Vis spectra were recorded using a UV/vis/NIR spectrophotometer to monitor the progress of Fe^3+^ to Fe^2+^ one-electron reduction reaction with light irradiation time.

### Electrochemical measurements

All the electrochemical measurements were performed in a three-electrode system on a biologic VSP-300 electrochemical workstation. Redox.me photo-electrochemical flow H-cell was used as the reactor for the electrochemical measurements. In a typical procedure, the working electrode was prepared by taking 5 mg of catalysts on an FTO plate, followed by adding 30 μL of 5 wt% Nafion solutions (Sigma-Aldrich) dropwise on the catalyst. The FTO plate was then dried in an open environment. Electrochemical impedance spectroscopy (EIS) measurements were conducted in 1 M KOH solution using Ag/AgCl (3 M KCl) electrode as the reference electrode, the platinum wire auxiliary electrode as the counter electrode, and the FTO electrode with catalyst as the working electrode. EIS measurements of the catalysts were carried out using the above three-electrode systems at 0.5 V vs Ag/AgCl. The frequency range was 100 kHz to 10 mHz, and the amplitude of the applied voltage was 10 mV.

### Kelvin probe force microscopy (KPFM) measurement

The KPFM was performed using Asylum/Oxford Instruments, MFP3D Origin. Using Au-evaporated film, the work function (WF) of the Ti/Ir tip was obtained as 4.96 eV. The Ni_3_N nanosheets sample for KPFM measurement was prepared by spin-coating Ni_3_N dispersion (3 mg of Ni_3_N nanosheets in 0.2 mL of ethanol) over an FTO-coated glass plate at 250 rpm for 1 s multiple times. The topology map and potential map of this sample were then obtained using a scan size of 1 μm at a scan rate of 0.75 Hz. During the scan, the light of wavelength 405 nm (using Thorlabs Diode Laser) was switched on and off. The work function in light off and light on of Ni_3_N nanosheets was calculated using the formula −eV_CPD_ = WF_tip_ − WF_sample_.

### Photoluminescence (PL) measurement

For the PL measurements, excitation was carried out at 630 nm using a diode laser. Methylene blue (MB) (250 μL of 2.5 mM) was taken in an Eppendorf, followed by adding 250 μL of DI water. Then, the resulting 0.5 mL solution (without Ni_3_N) was taken in the cuvette and excited at 630 nm to record its PL. Then the emission of MB was measured in the presence of Ni_3_N nanosheets. For that, 250 μL of 2.5 mM MB was taken in an Eppendorf, followed by the addition of 250 μL of Ni_3_N nanosheets dispersion (0.5 mg mL^−1^ in DI water). The resulting dispersion (0.5 mL) was taken in the cuvette and excited at 630 nm to record its emission. The photoluminescence of pure Ni_3_N nanosheets was measured by taking 250 μL of dispersion and 250 μL of DI water followed by excitation at 630 nm.

### AFM measurement

The thickness of the Ni_3_N nanosheets was measured by Park XE-70 AFM. For measurement, Ni_3_N nanosheets were drop-cast on an optically flat silicon wafer and placed under the AFM tip for imaging. The measurement was done in contact mode.

### Photocurrent measurement

Photocurrent measurement was carried out using a custom-made two-probe system and Keithley 2602 source meter. The measurement was carried out in the dark and under light illumination using a 300 W Xenon Lamp (0.5 W cm^−2^, 400–1100 nm).

### Finite-difference time-domain (FDTD) simulations

The electric field enhancement calculations were performed by the finite-difference time-domain method. For simulation, the Ni_3_N nanosheets were modeled as 10-nm-thick sheets of 50 × 50 nm dimension. An x and y-polarized total-field scattered-field (TFSF) source having a wavelength range of 400–1100 nm and E_0_ of 16.6 V m^−1^ was used as the excitation source to mimic the photocatalysis conditions. Frequency domain field profile monitors were used to calculate the electric field distribution in all the simulations. The dielectric constants of Ni_3_N were not known, and hence we used the dielectric constant of ZrN to estimate the electric field in Ni_3_N.

### Competitive CO_2_ and methyl-p-benzoquinone reaction by hot electrons

During the photocatalytic CO_2_ hydrogenation reaction, 50 μL of 1 M solution of Methyl-p-benzoquinone (MBQ) in diethyl ether was quickly added to the catalyst bed (after the pretreatment step) by opening the reaction chamber under 100 mL min^−1^ argon flow. The reactor was then heated at 50 °C for 1 h under argon (150 mL min^−1^) to remove the solvent. The reactant gases were introduced into the reactor chamber through Alicat mass flow controllers; CO_2_ at 73 mL min^−1^, H_2_ at 4 mL min^−1^, with a total flow of 77 mL min^−1^ at 1 bar pressure. The catalyst was then irradiated with light (300 W Xenon Lamp ~2.5 W cm^−2^, 400–1100 nm), and the reaction’s progress was monitored using an online MicroGC. MBQ was added again by the same protocol, and the photocatalytic CO_2_ hydrogenation reaction was then monitored under light irradiation.

### In situ DRIFTS study for CO_2_ hydrogenation

A diffuse reflectance infrared Fourier transform spectroscopy (DRIFTS) measurement was carried out to understand the reaction mechanism of photocatalytic CO_2_ hydrogenation over Ni_3_N nanosheet. DRIFTS measurements were performed using JASCO FT/IR-4700 instrument, with a DiffusIR™-PIKE Technologies reaction chamber with KBr windows. This catalyst (8.2 mg) was taken in a porous ceramic cup inside the reaction chamber. DRIFTS measurements were conducted after the catalyst was pretreated at 150 °C in Argon flow of 100 mL min^−1^, and a baseline was taken in Ar flow of 19 mL min^−1^ with 7200 scans and 4 cm^−1^ resolutions. The reactant gases were introduced through Alicat mass flow controllers; CO_2_ at 18 mL min^−1^, H_2_ at 1 mL min^−1^, with a total flow of 19 mL min^−1^. The catalyst was exposed to light (Xenon Lamp 400–1100 nm). After 5 min, we started to record the DRIFT spectra with 7200 scans and 4 cm^−1^ resolution under continuous gas flow and light exposure. For DRIFTS in dark conditions, we performed the experiment using exactly the same conditions (described above) but without light and with external heating. For DRIFTS in only CO_2_ conditions, we performed the experiment using exactly the same conditions without H_2_ with light and without external heating.

### Nanosecond time-resolved measurements

The nanosecond transient absorption and emission measurements were carried out on a setup previously reported^1^. The excitation source was an EKSPLA Model NT342B, consisting of an Nd:YAG rod with a 1064 nm output that was frequency tripled to 355 nm before being redirected to pump the OPO (Optical Parametric Amplifier) consisting of type II nonlinear BBO crystals, which allowed for facile wavelength tuning in the visible range (410–710 nm) using the associated software (L900) on the connected computer. The pump wavelength chosen was 500 nm and was attenuated with the help of neutral density filters at the output port to furnish a power of 10 mJ/pulse at the sample (repetition rate: 10 Hz). The setup utilized a right-angle detection geometry, and an arced Xenon lamp served as the (white) probe light source; both pump and probe were pulsed for the transient absorption measurements to improve signal-to-noise, while the emission measurements used the pump only.

The detection system used was LP920 (Edinburgh Instruments) equipped with a photomultiplier tube and an Andor iStar CCD camera cooled to −8 °C for acquiring kinetic and spectral data, respectively, and the data acquisition frequency was 1 Hz. The L900 software also controlled the detection system on the connected computer for data collection. All measurements utilized a slit-width corresponding to a spectral resolution of 5 nm. For the spectral measurements, the central wavelength (monochromator) of the CCD camera was positioned at 575 nm, the counts were typically around 40,000, and the gate width (integration time) was held fixed at 20 ns. Every measurement was averaged over 16 shots. The kinetic data were fit with the help of the in-built iterative fitting routine in L900 and plotted using MATLAB R2020b, with the smooth function using a span of 20.

## Supplementary information

Supplementary Information

Description of Additional Supplementary Files

Supplementary Data 1

Supplementary Data 2

## Data Availability

The data that support the findings of this work are available within the article and its supplementary information. Source data are provided with this paper.

## Source data

Source Data
