# Supplementary material for: Surface plasmon-enhanced photo-driven CO2 hydrogenation by hydroxy-terminated nickel nitride nanosheets
Source: Nat Commun. 2023 May 3;14:2551. doi: 10.1038/s41467-023-38235-9 (PMC10156734; doi:10.1038/s41467-023-38235-9)
Supplement: Supplementary file 1 — Supplementary Information [file 41467_2023_38235_MOESM1_ESM.pdf]

Supplementary Information

Surface plasmon-enhanced photo-driven CO_2 hydrogenation by hydroxy-terminated nickel nitride nanosheets

Saideep Singh,[1#] Rishi Verma, [1#] Nidhi Kaul, [2#] Jacinto Sa,[2] Ajinkya Punjal, [3] Shriganesh Prabhu,[3] and Vivek Polshettiwar[1]*

[1]Department of Chemical Sciences, Tata Institute of Fundamental Research, Mumbai, India
[2]Department of Chemistry-Ångström Laboratory, Uppsala University, Uppsala, Sweden
[3]Department of Condensed Matter Physics and Materials Science, Tata Institute of Fundamental Research, Mumbai, India
*Corresponding author. Email- vivekpol@tifr.res.in # equally contributing authors

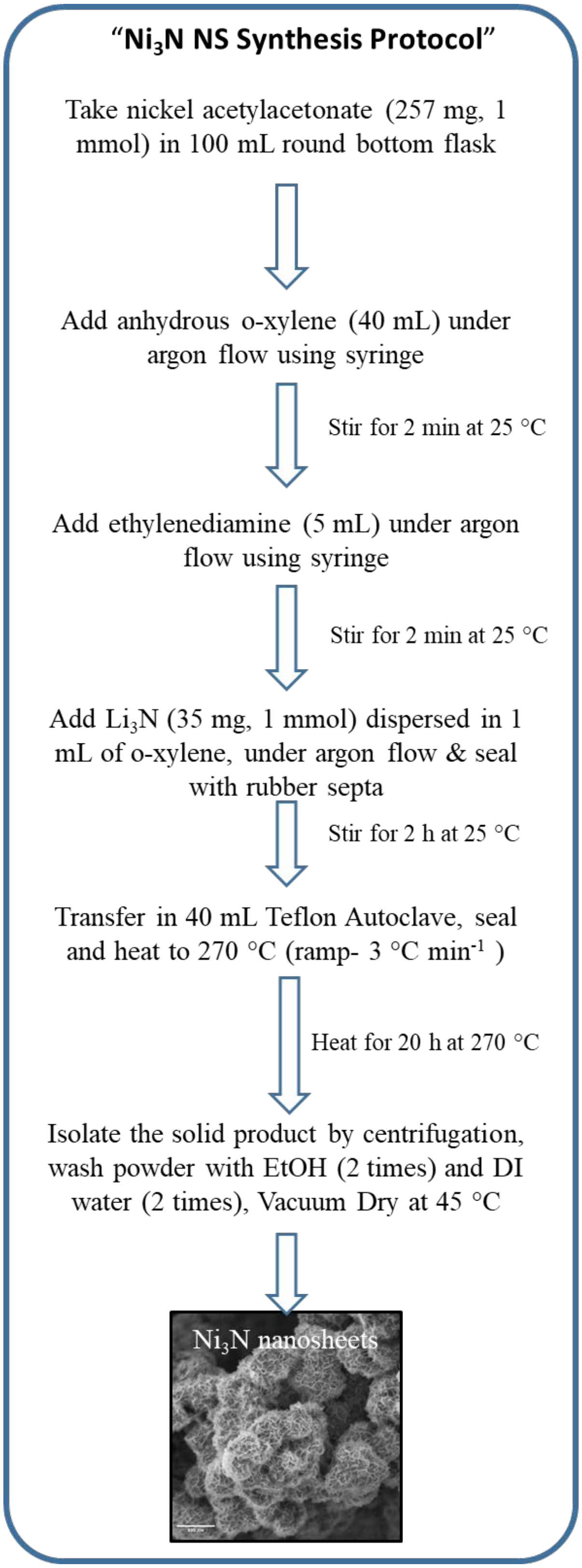

Figure S1. Schematic of synthesis of Ni_3N nanosheets.

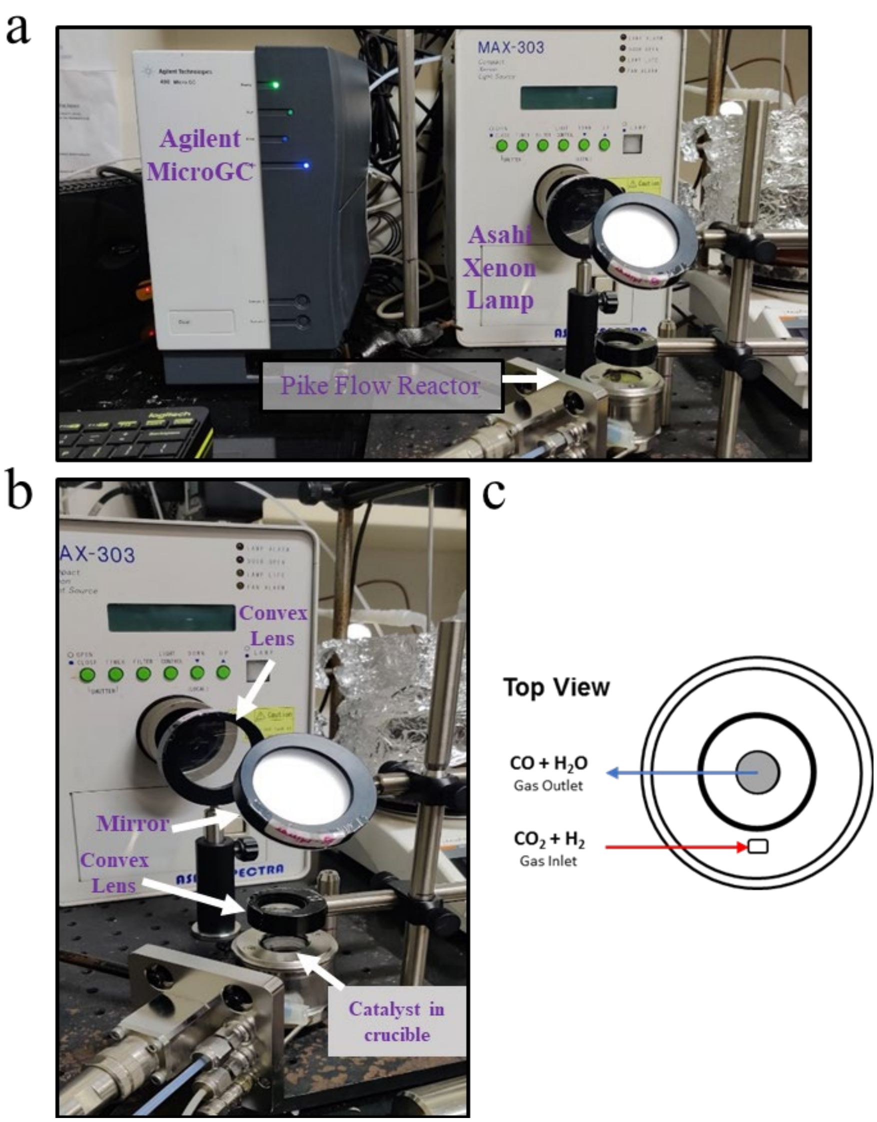

Figure S2. Photocatalytic CO_2 hydrogenation in light and dark experimental setup, (a, b) Photograph of the experimental setup, and (c) interaction of gas flow with the catalyst powder in the crucible.

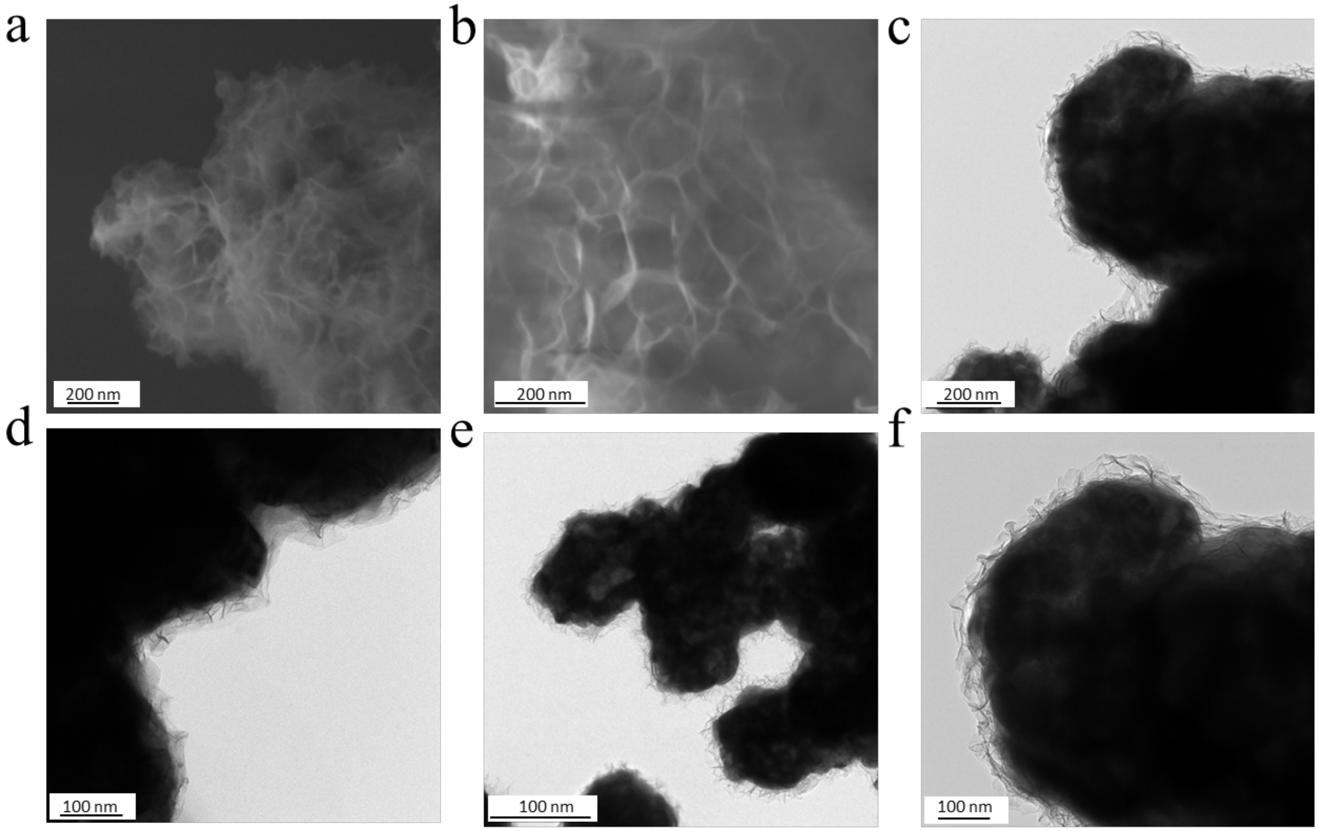

Figure S3. (a,b) SEM and (c-f) TEM images of Ni_3N nanosheets.

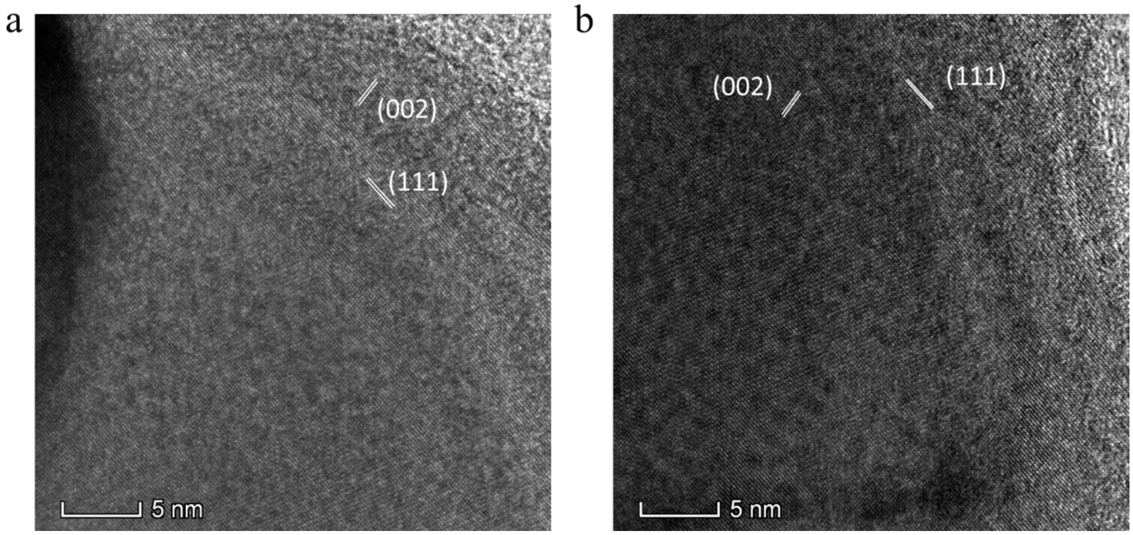

Figure S4. HRTEM images of Ni_3N nanosheets.

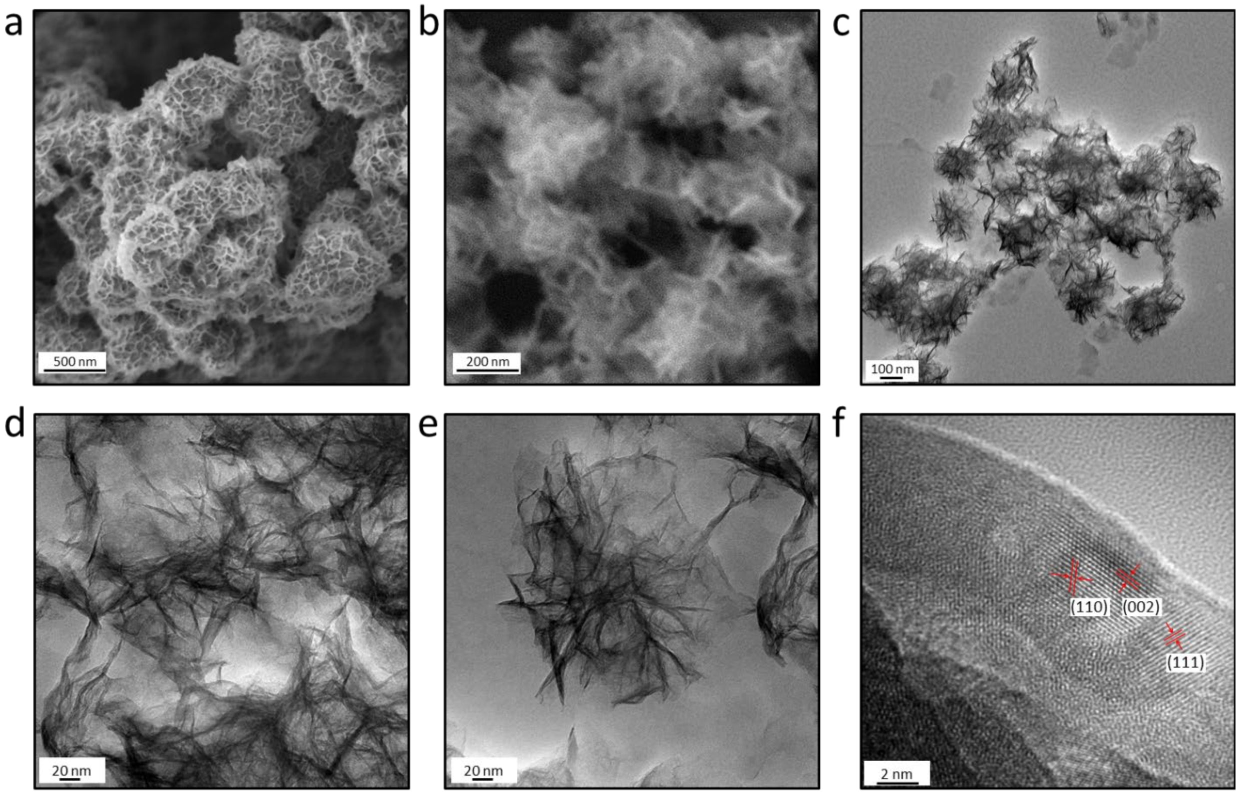

Figure S5. Additional (a,b) SEM images; (c-f) HRTEM images of Ni_3N nanosheets.

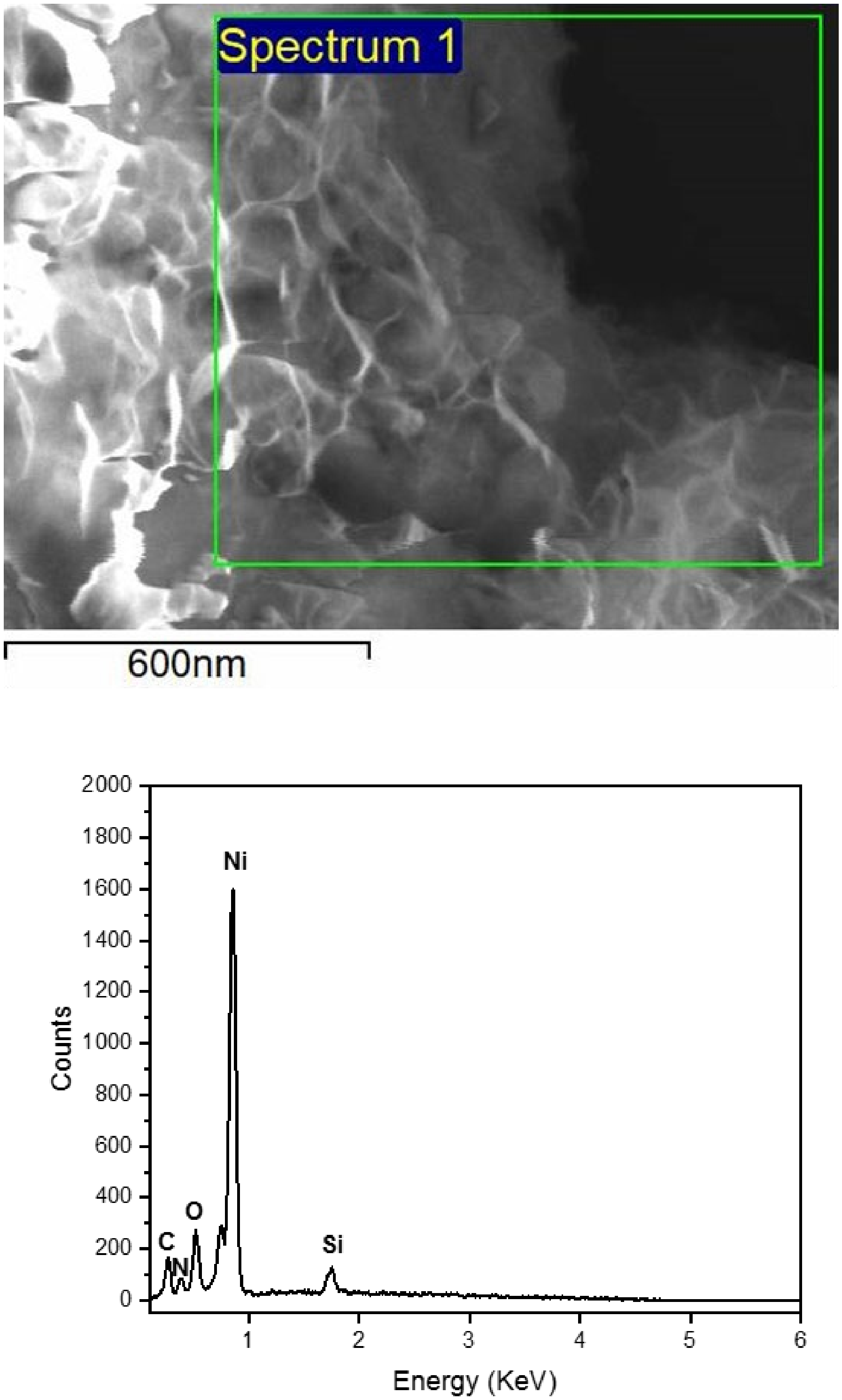

Figure S6. EDS spectrum of Ni_3N nanosheets (bottom) and respective SEM image (top).

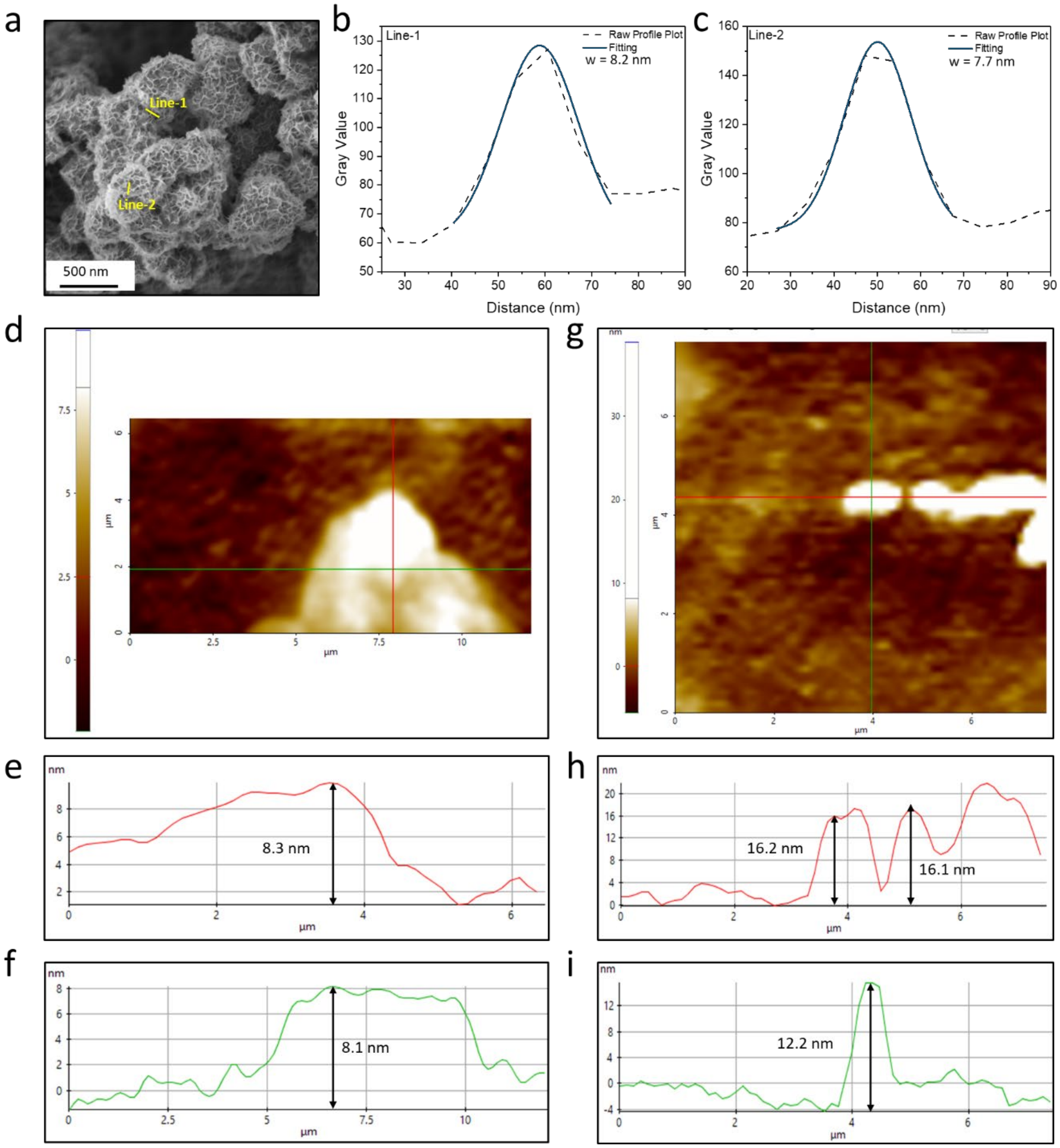

Figure S7. (a) SEM image and (b,c) line profile plots of line-1 and line-2 in a; (d,g) AFM images and (e,f,h,i) respective height profiles along the red and green lines. e & f for image d, and h & i for image g.

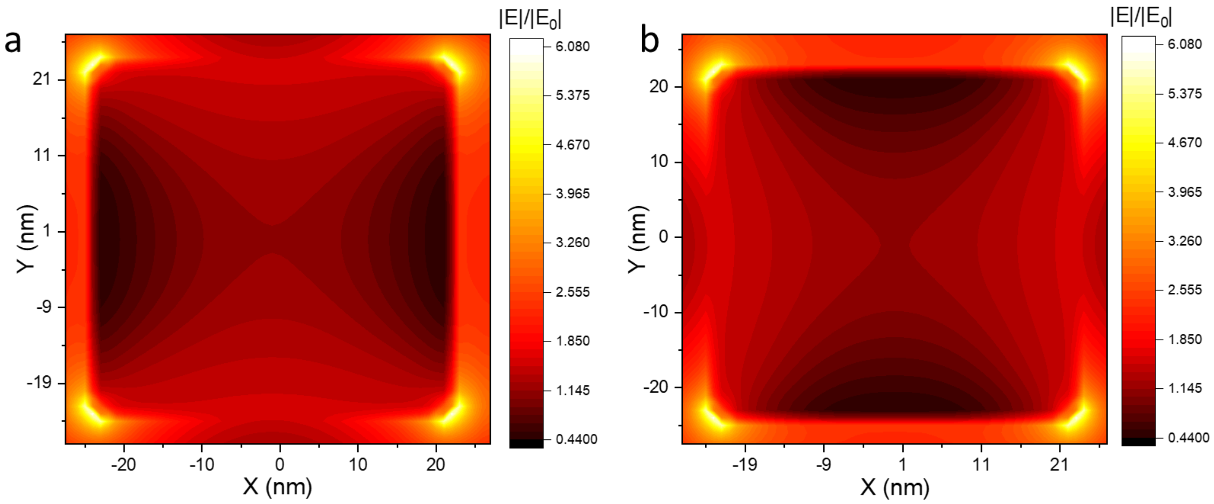

Figure S8. Electric field enhancement in Ni_3N nanosheets by (a) y-polarized and (b) x-polarized light excitation (400-1100 nm), using finite-difference time-domain (FDTD) simulation. The dielectric constants of Ni_3N was not known and hence we used the dielectric constant of ZrN (Adv. Photonics Res. 2021, 2, 2100178) to estimate the electric field in Ni_3N.

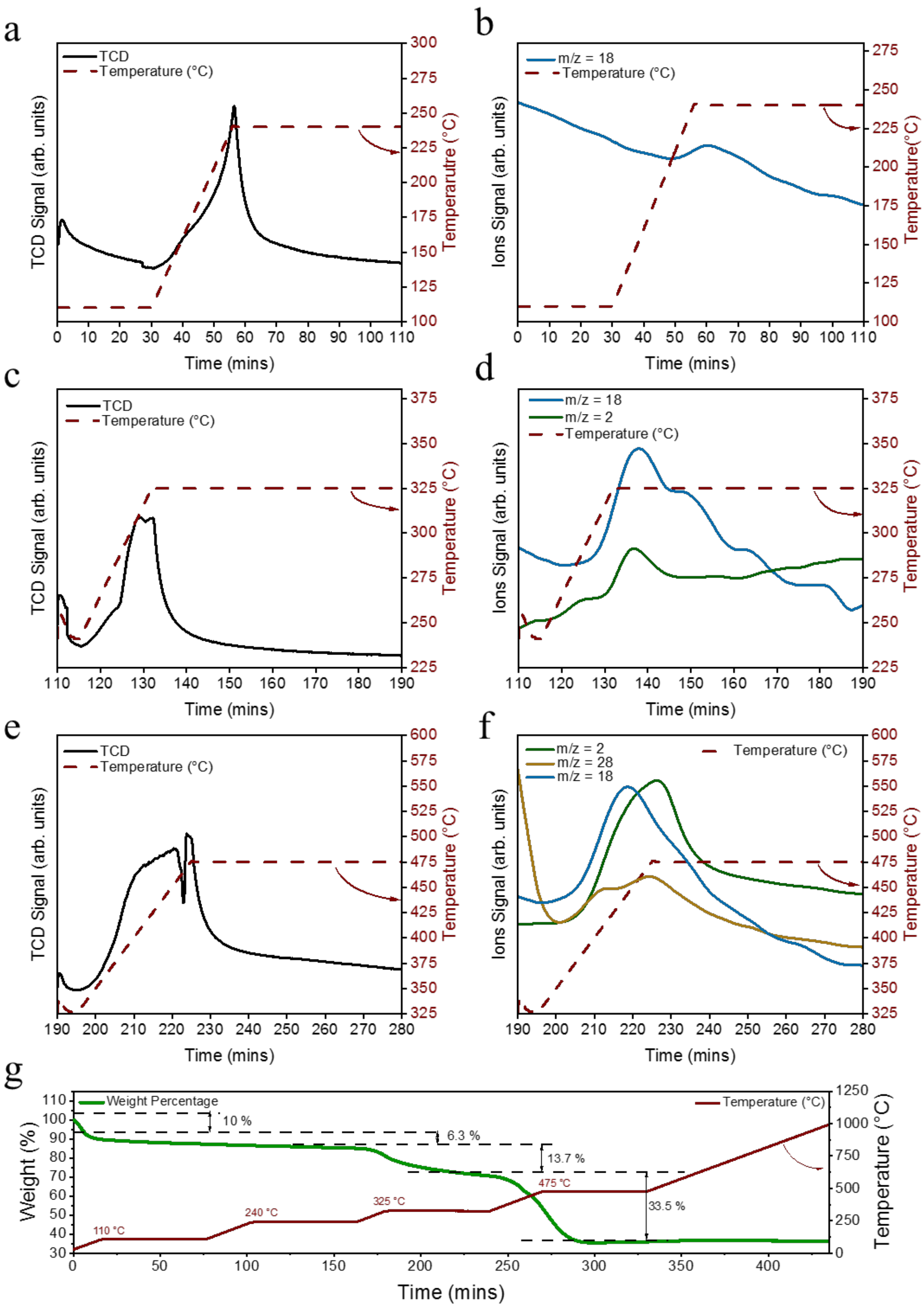

Figure S9. Temperature Programmed Reaction (TPReaction) of Ni_3N nanosheets. (a,c,e) TCD signal and (b,d,f) mass spectra of various products, formed at different temperature ranges, (a,b) 120 – 240 °C, (c-d) 240 – 325 °C, (e-f) 325- 475 °C; (g) Thermogravimetric analysis (TGA) of Ni_3N nanosheets.

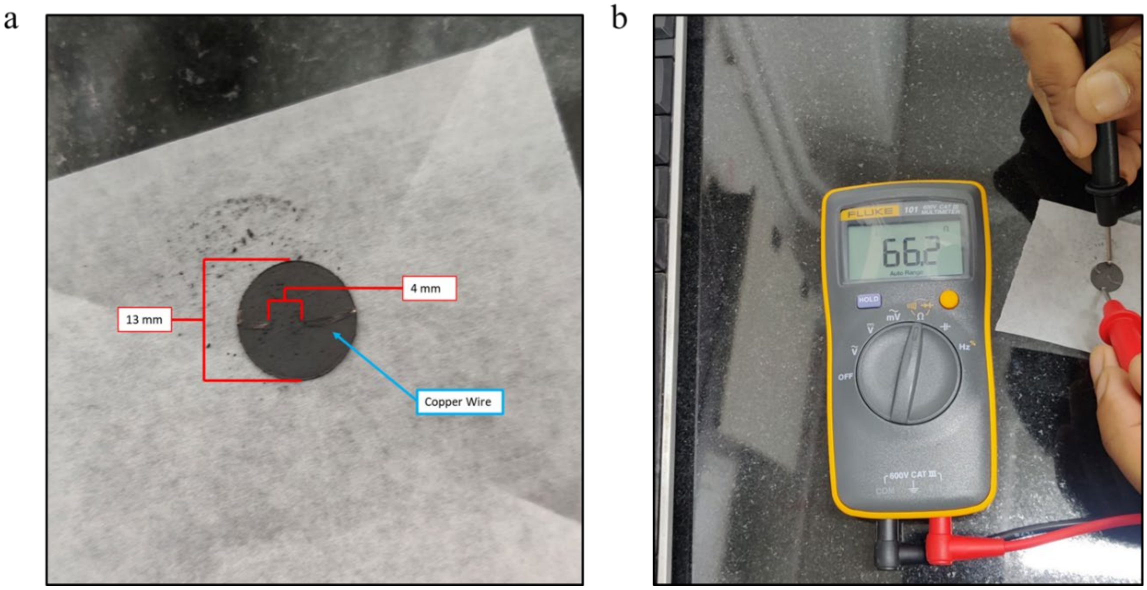

Figure S10. Measurement of electrical resistivity of Ni_3N nanosheet. (a) Ni_3N pellet, (b) resistivity measurement by fluke multimeter.

Calculations:

$$\text{Resistivity Formula:} = \frac{Resistance(ohm) \times Area(m^2)}{length(m)}$$

$$= \frac{66.2 \times 3.14 \times (0.1 \times 10^{-3})^2}{(13 \times 10^{-3})}$$

$$= \frac{207.8 \times 10^{-8}}{13 \times 10^{-3}}$$

$$= 16.0 \times 10^{-5}\ ohm\ m$$

$$\text{Conductivity} = \frac{1}{Resistivity}$$

$$= \frac{1}{16.0 \times 10^{-5}\ ohm\ m} = \mathbf{6.25 \times 10^{3}\ S\ m^{-1}}$$

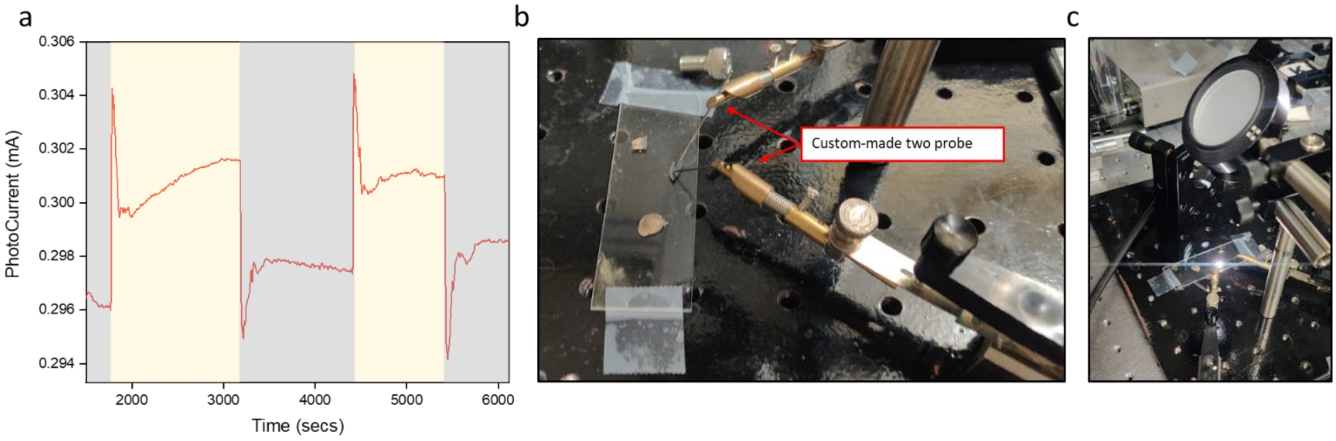

Figure S11. (a) Photocurrent measurement of a Ni_3N nanosheet in a light on-off cycle using a xenon lamp (400- 1100 nm) under an external bias of 100 mV, (b,c) experimental setup for photocurrent measurement.

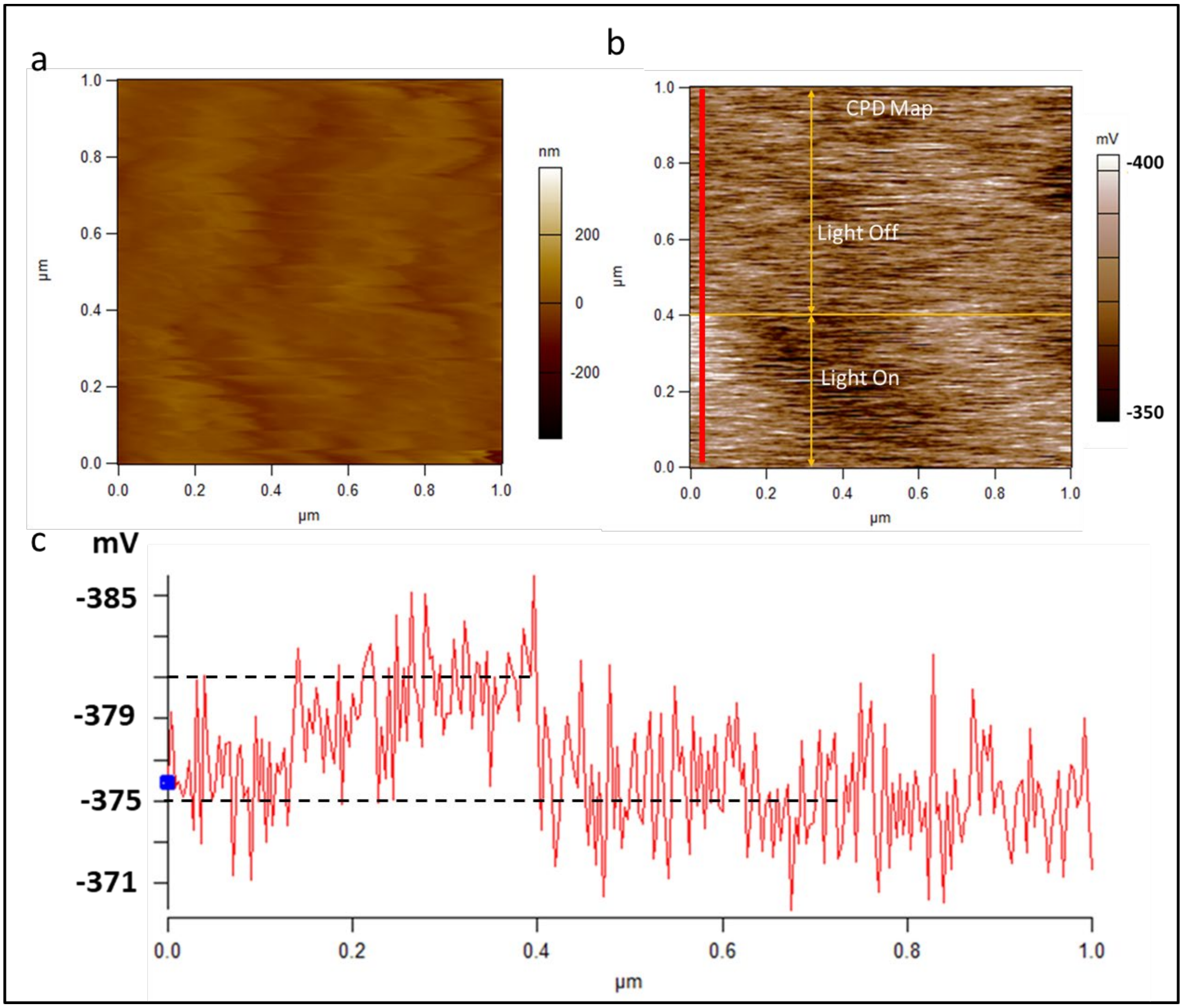

Figure S12. (a) AFM height profile image, (b) KPFM potential image of the Ni_3N nanosheets, (c) Line map (shown in red) of potential from image (b).

The calculated work function of Ti/Ir tip is 4.96 eV

$$Work\ Function\ in\ dark\ (WF_{dark}):\ \varphi_D = (4.96 - 0.375)\ eV = 4.585\ eV$$

$$Work\ Function\ in\ light\ (WF_{light}):\ \varphi_L = (4.96 - 0.381)\ eV = 4.579\ eV$$

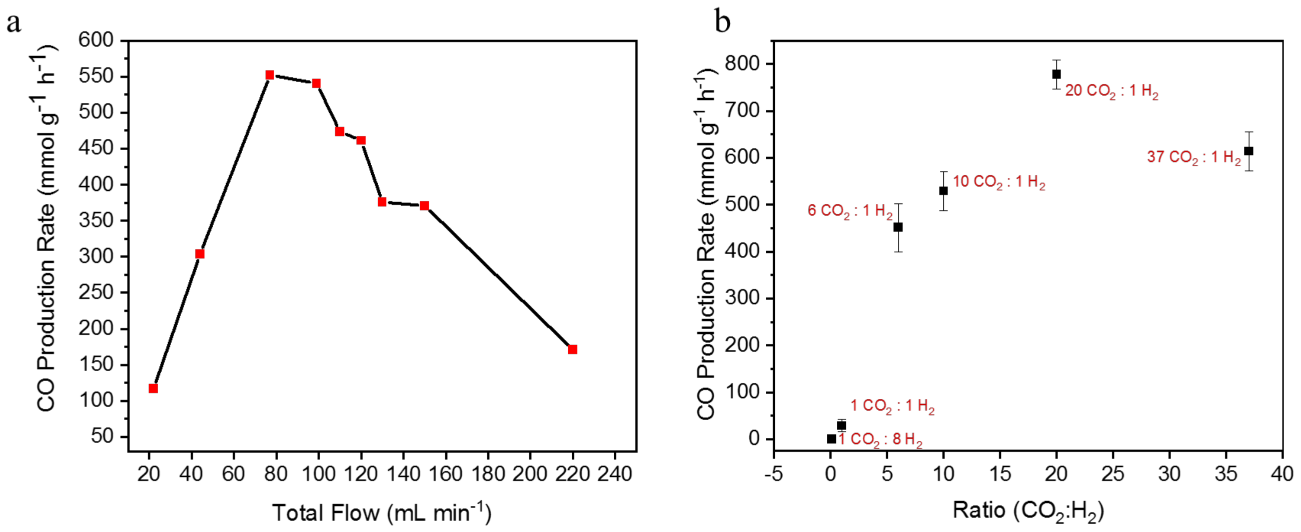

Figure S13. Photocatalytic CO production rate by Ni_3N nanosheets as a function of (a) total flow and (b) ratio of CO_2:H_2 at a total flow of 77 mL min^{-1}. Error bars: Calculated from data of at least three repeated experiments.

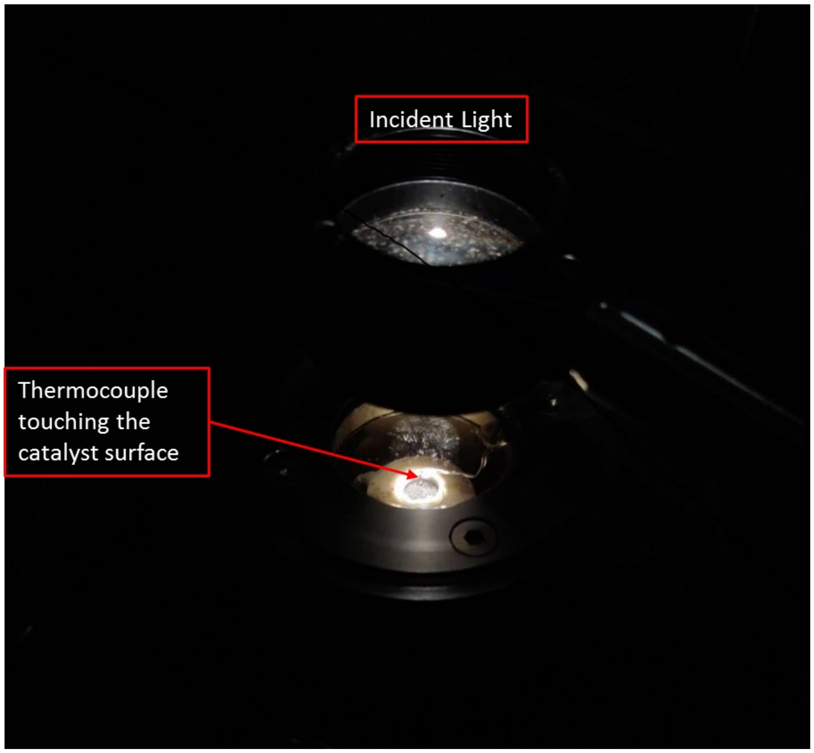

Figure S14. Setup for measuring the catalyst bed temperature using thermocouple touching the catalyst surface, irradiated with 400-1100 nm light using xenon lamp at 2.5 W cm^{-2}.

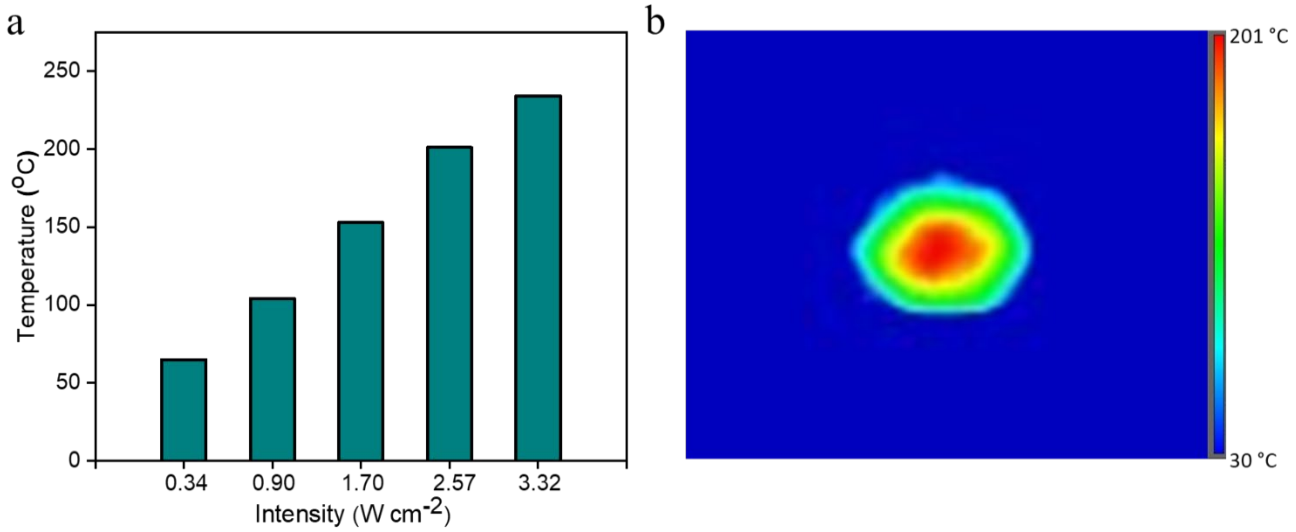

Figure S15. (a) Temperature as a function of the intensity measured by IR thermal imaging camera (FLIR A6700sc), (b) temperature profile of the sample having 1 cm diameter irradiated with 400-1100 nm light using xenon lamp at 2.5 W cm $^{-2}$.

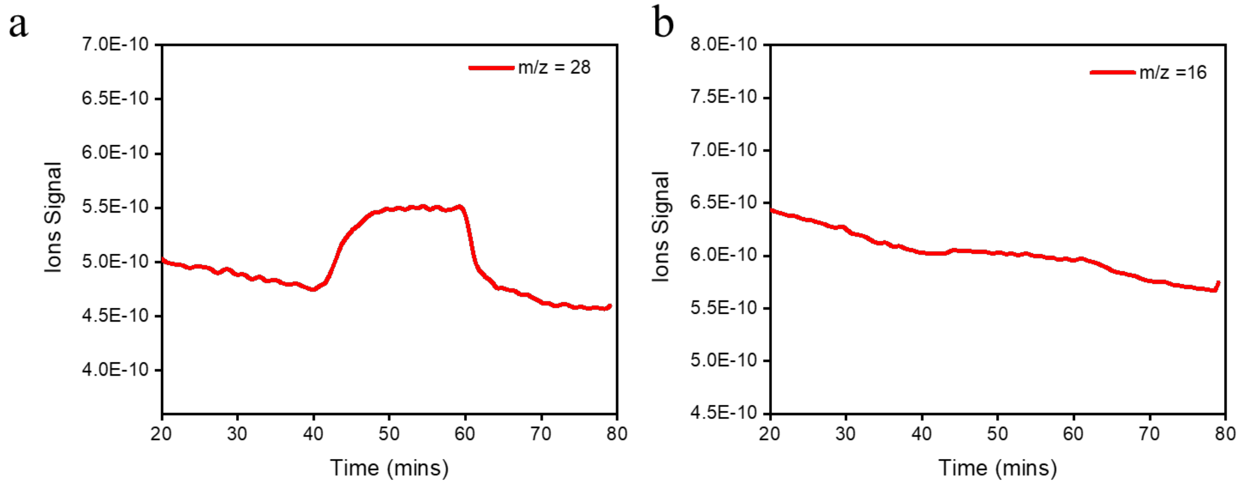

Figure S16. Ion counts of products of photocatalytic CO_2 hydrogenation in a mass spectrometer (MS), (a) CO, (b) CH_4.

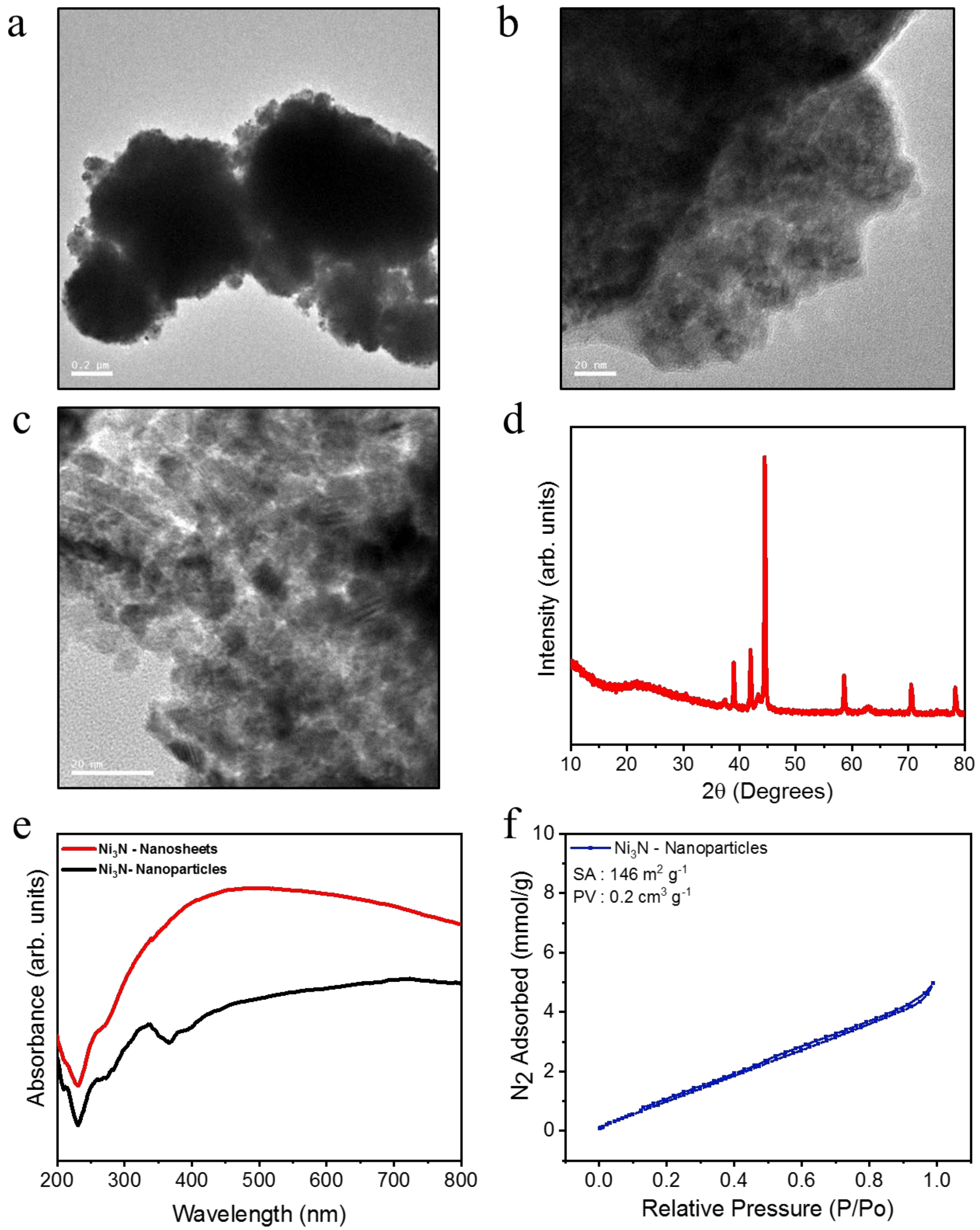

Figure S17. Characterization of Ni_3N nanoparticles, (a-c) TEM images of Ni_3N nanoparticles, (d) PXRD pattern, (e) absorption spectra of Ni_3N nanosheets and Ni_3N nanoparticle, (f) N_2 sorption isotherm of Ni_3N nanoparticles.

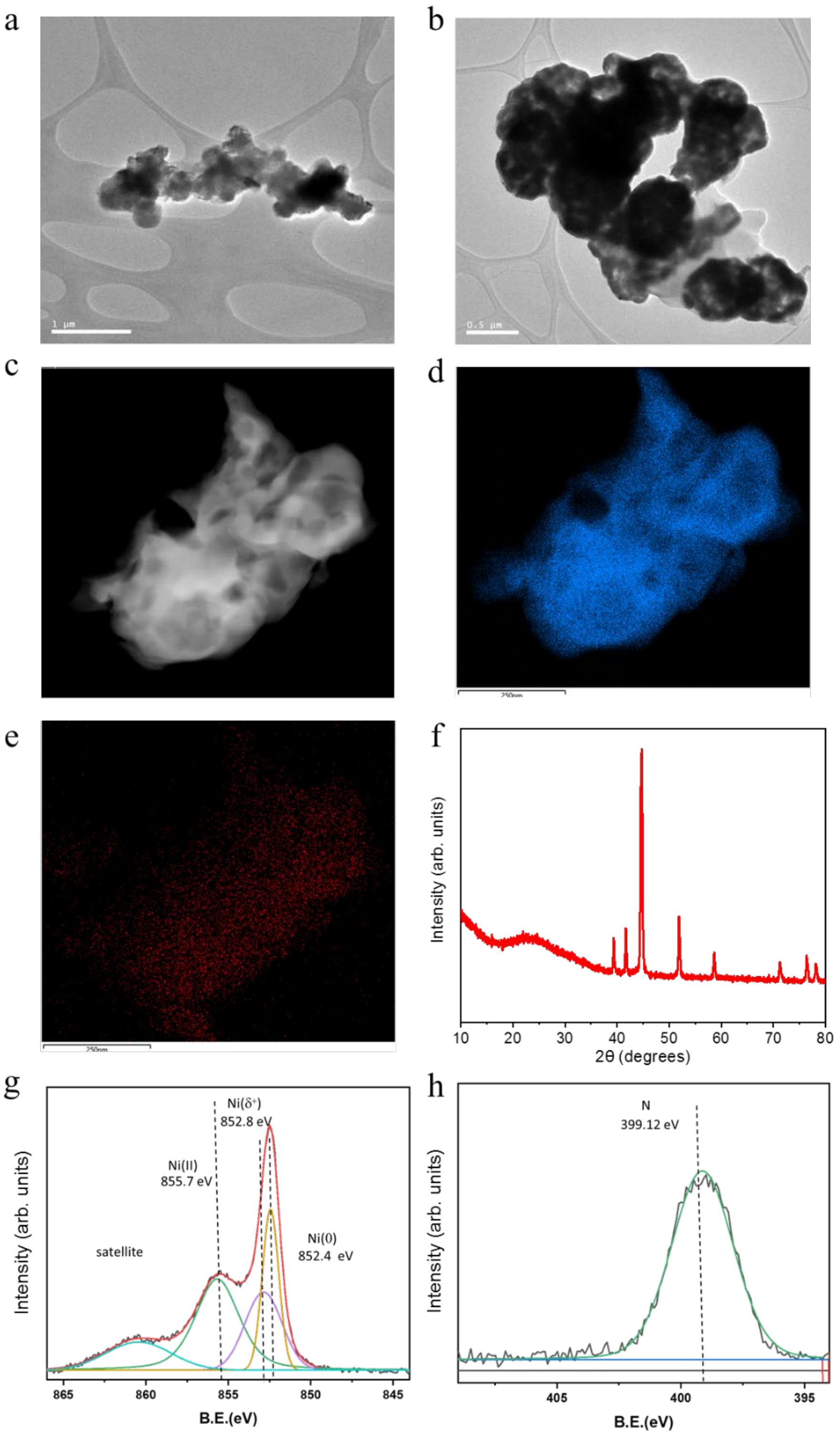

Figure S18. Characterization of Ni_3N nanosheets after photocatalytic CO_2 hydrogenation, (a,b) TEM images, (c) Dark field image of Ni_3N nanosheets and corresponding EDS mapping (d) Ni and (e) N, (f) PXRD pattern, XPS spectra expanded in the region of (g) Ni $2p_{3/2}$, and (h) N $1s$.

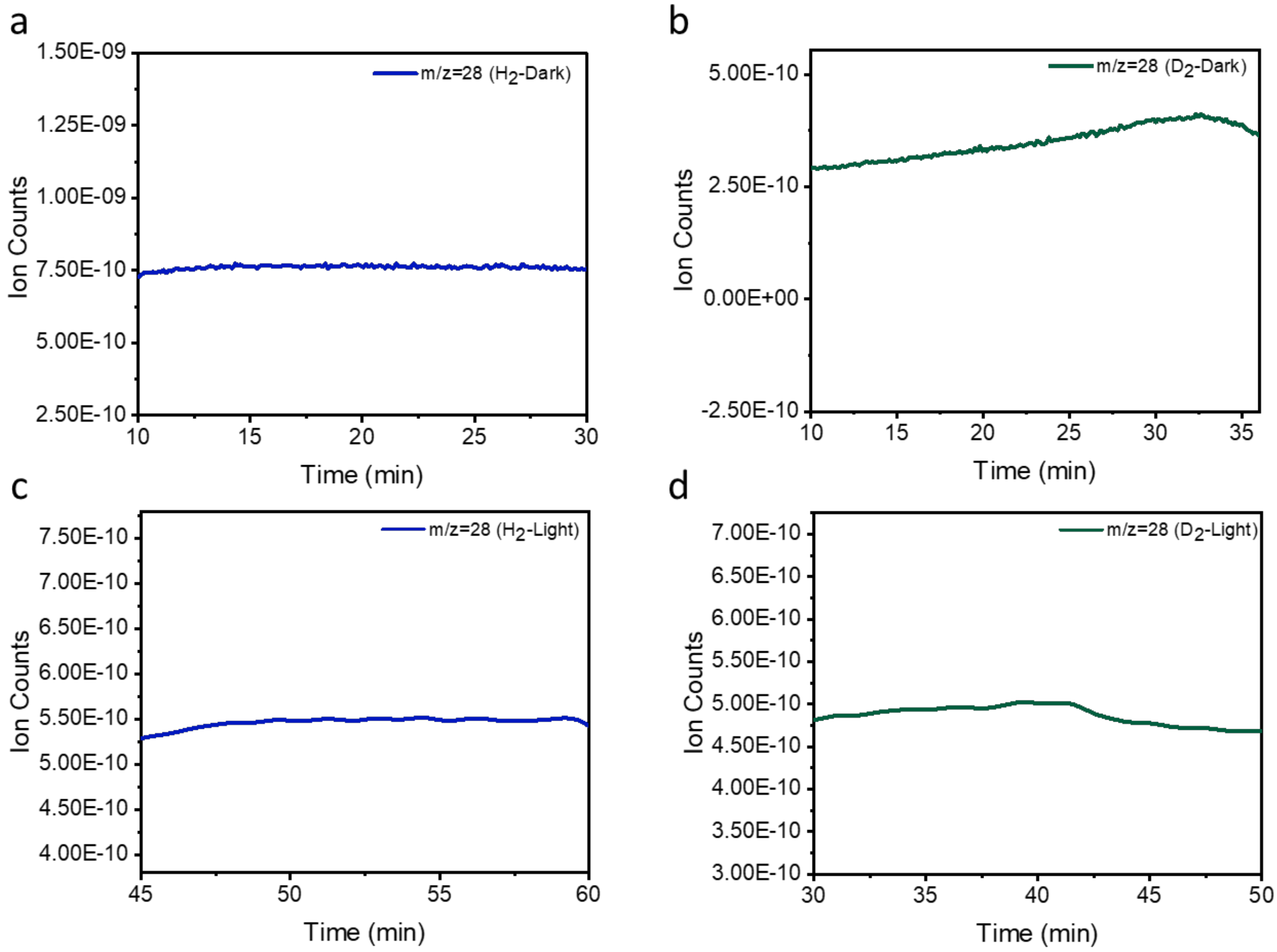

Figure S19. Ions signal of CO during Ni_3N nanosheets catalyzed reaction of (a) $CO_2 + H_2$, (b) $CO_2 + D_2$ in dark and (c) $CO_2 + H_2$, (d) $CO_2 + D_2$ in light.

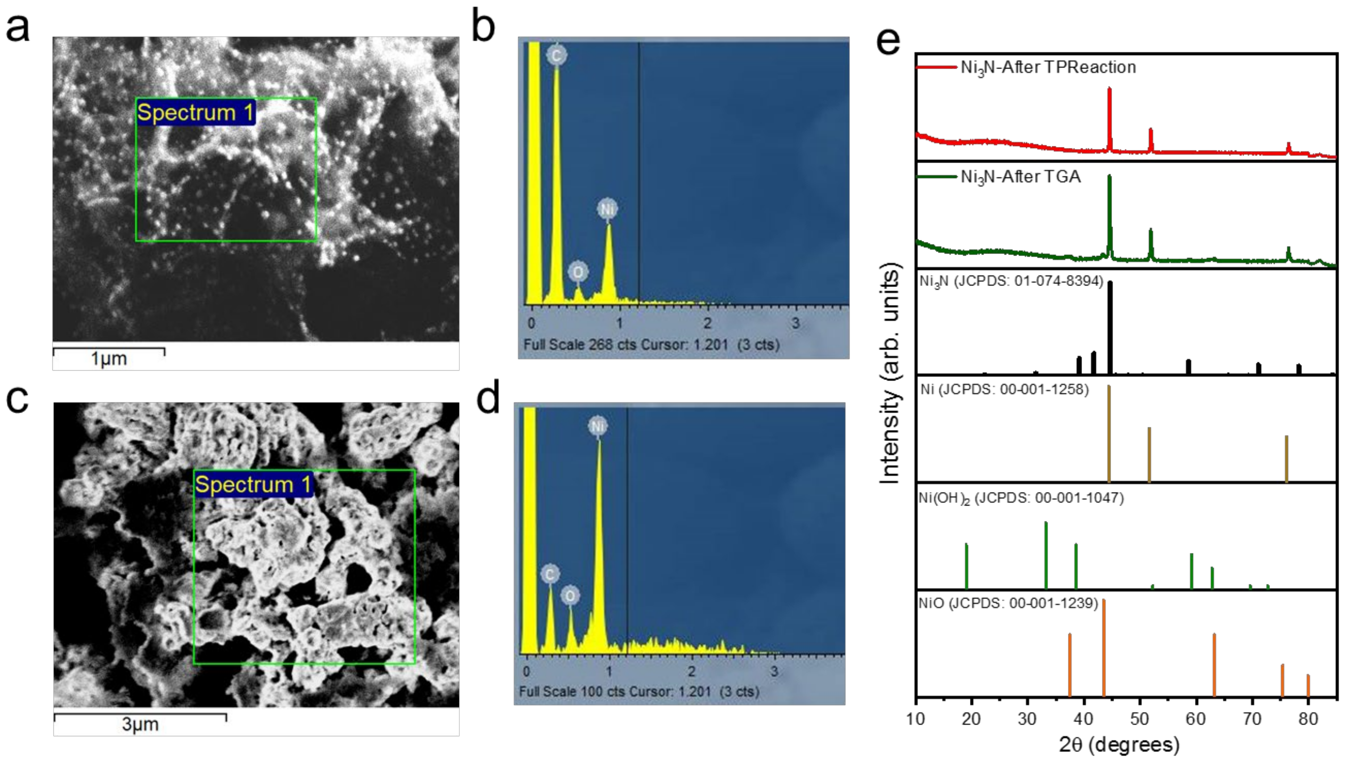

Figure S20. Characterization of Ni_3N nanosheets, (a) SEM, (b) EDS spectrum after TPReaction, and (c) SEM, (d) EDS spectrum after TGA; (e) PXRD of Ni_3N after TPReaction and TGA.

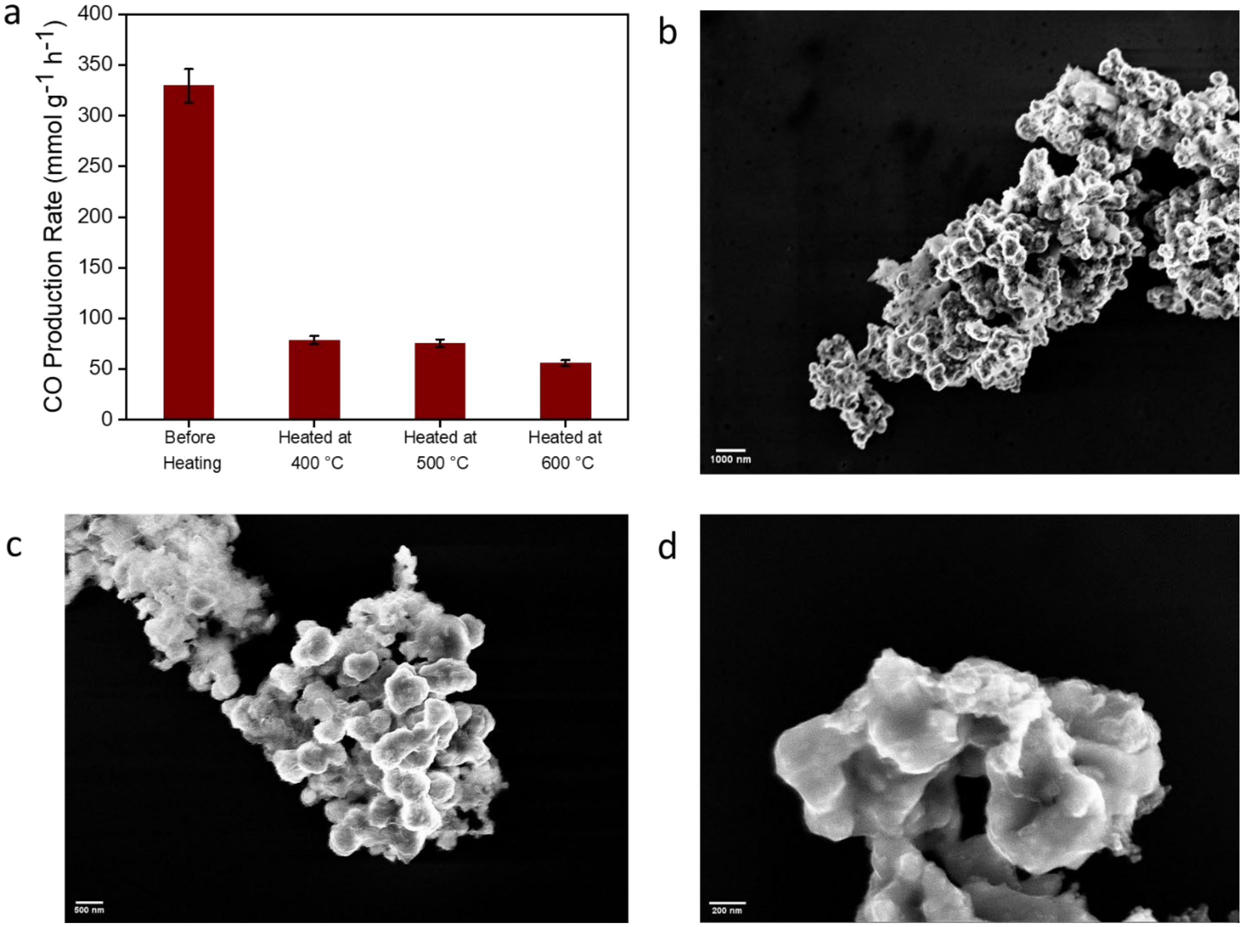

Figure S21. (a) Photocatalytic CO_2 hydrogenation by Ni_3N nanosheets after treating it at different temperatures. SEM images of Ni_3N nanosheets heated at (b) 400 ºC, (c) 500 ºC, and (d) 600 ºC. Error bars: Calculated from data of at least three repeated experiments.

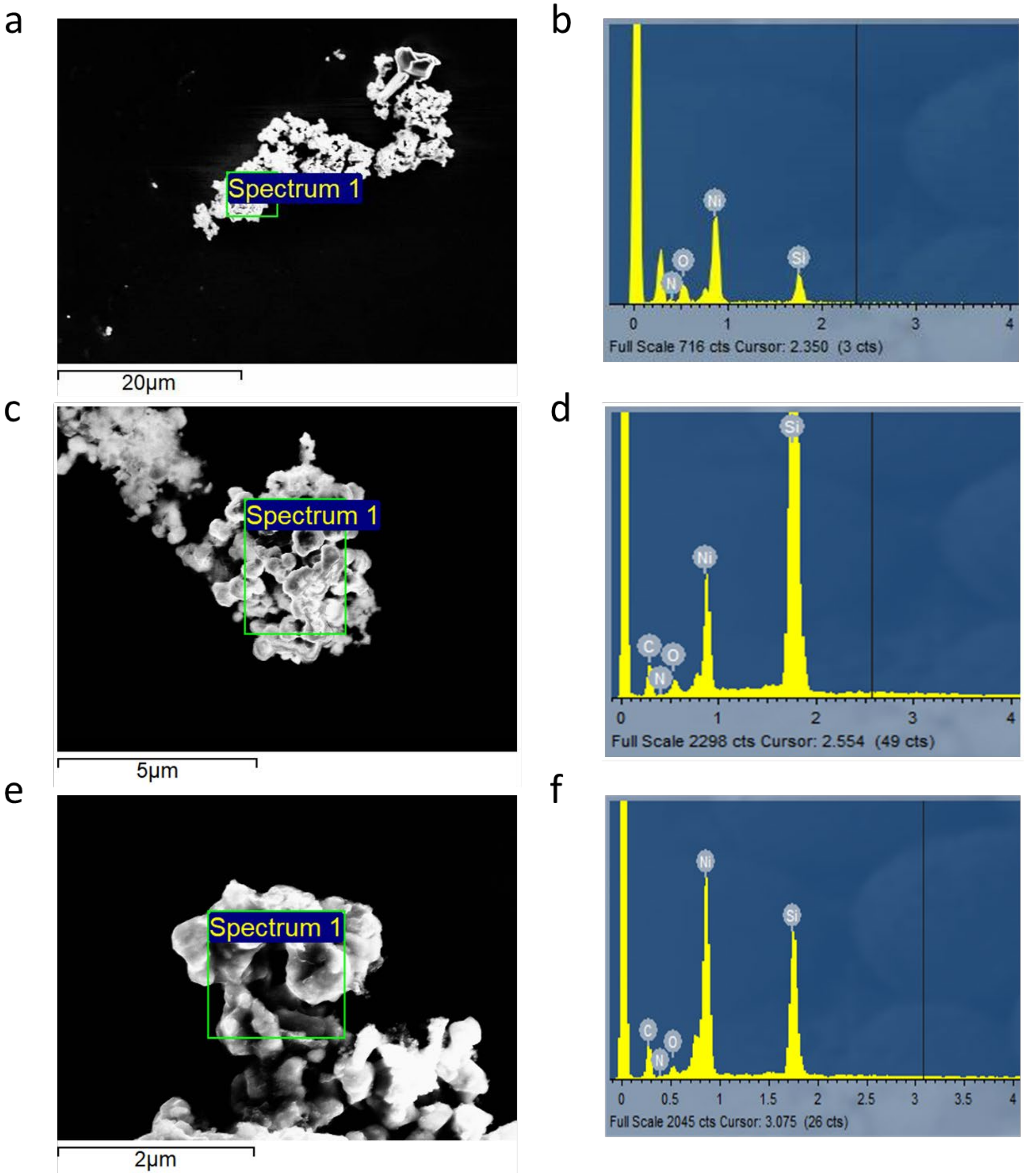

Figure S22. SEM images and EDS of Ni_3N nanosheets heated at (a,b) 400 ºC, (c,d) 500 ºC, and (e,f) 600 ºC, for 1 h in argon atmosphere.

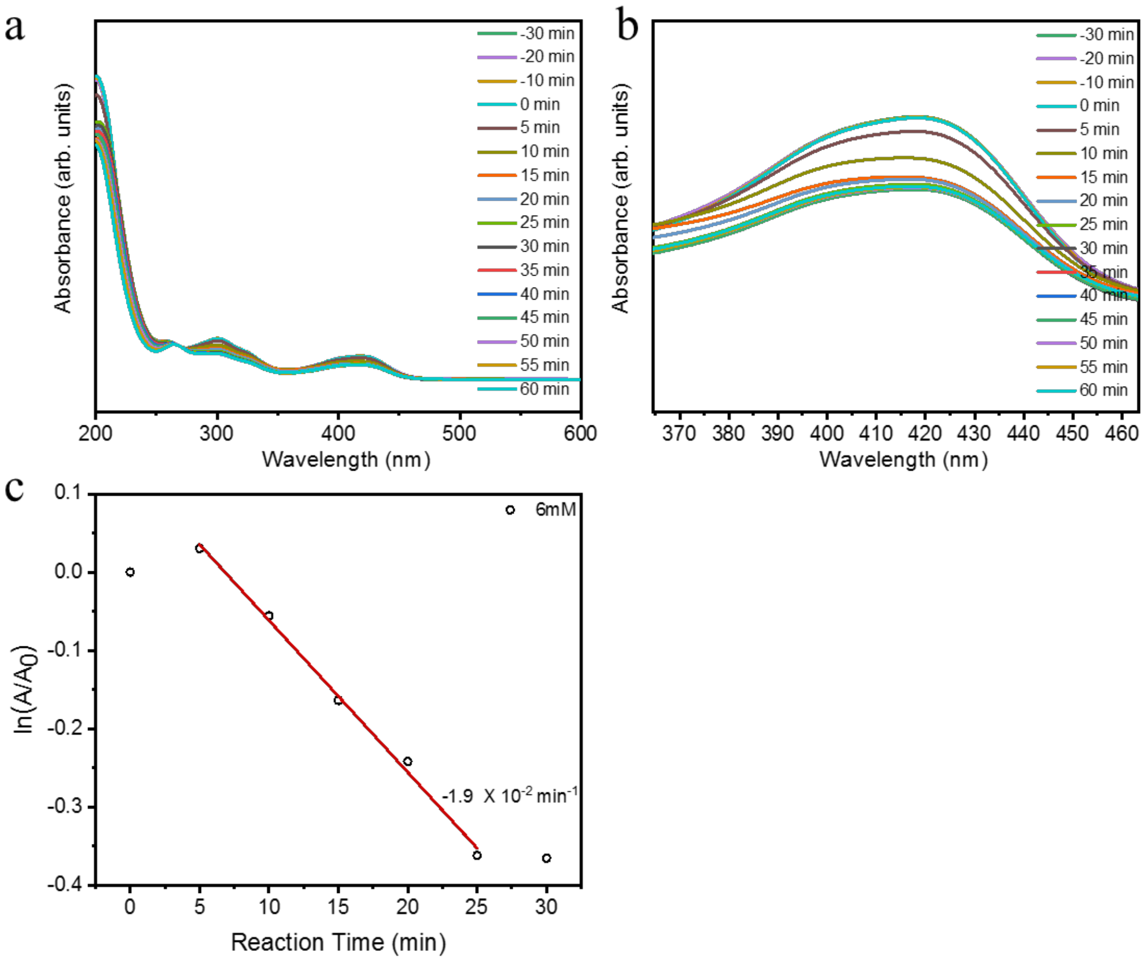

Figure S23. (a) UV-Vis spectra showing the conversion of Fe^{3+} to Fe^{2+} as a function of irradiation time for Ni_3N using 6 mM $K_3[Fe(CN)_6]$. (b) Magnified UV-Vis spectra around 419 nm. (c) Pseudo-first-order plot of $\ln(A/A_0)$ against reaction time for Ni_3N.

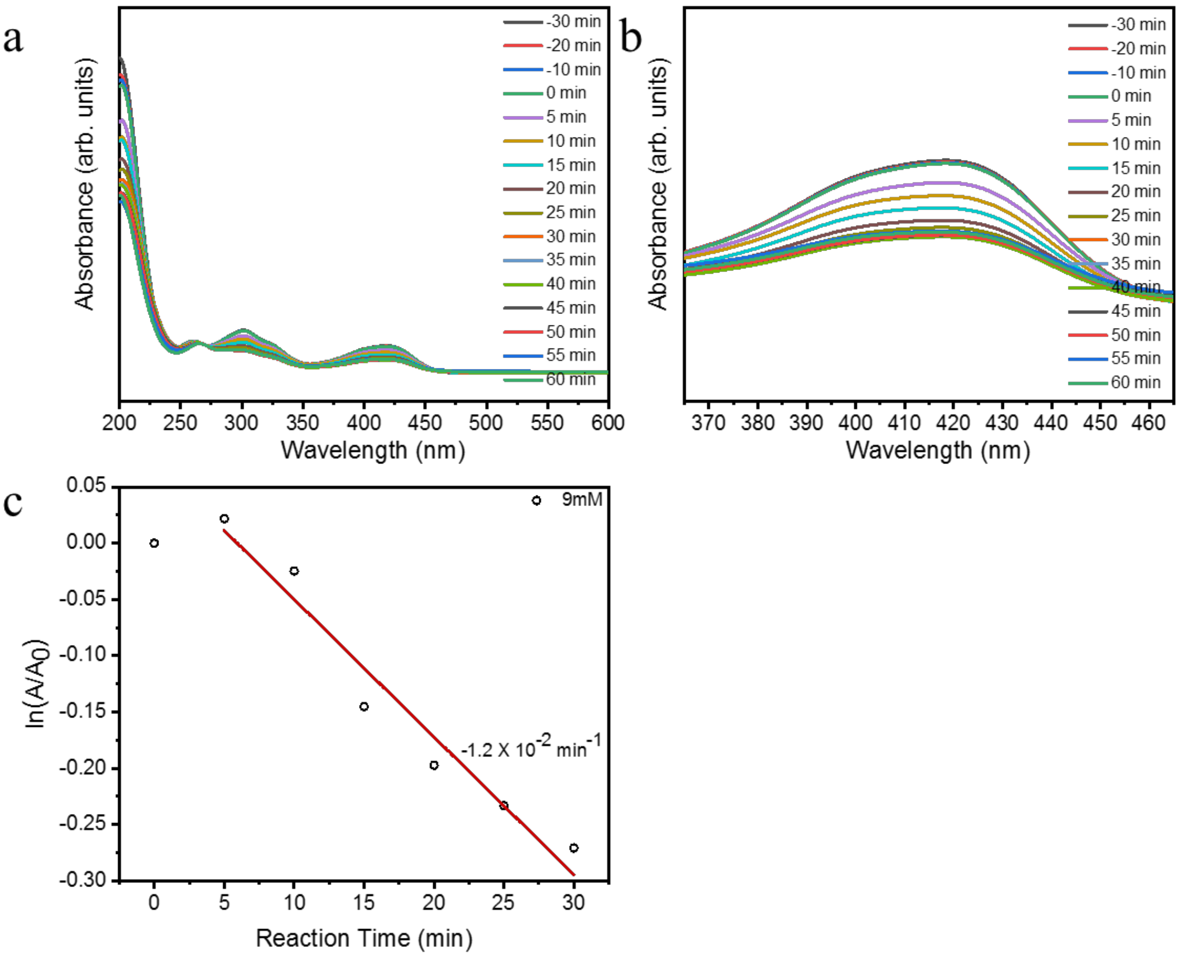

Figure S24. (a) UV-Vis spectra showing the conversion of Fe^{3+} to Fe^{2+} as a function of irradiation time for Ni_3N using 9 mM $K_3[Fe(CN)_6]$. (b) Magnified UV-Vis spectra around 419 nm. (c) Pseudo-first-order plot of $\ln(A/A_0)$ against reaction time for Ni_3N.

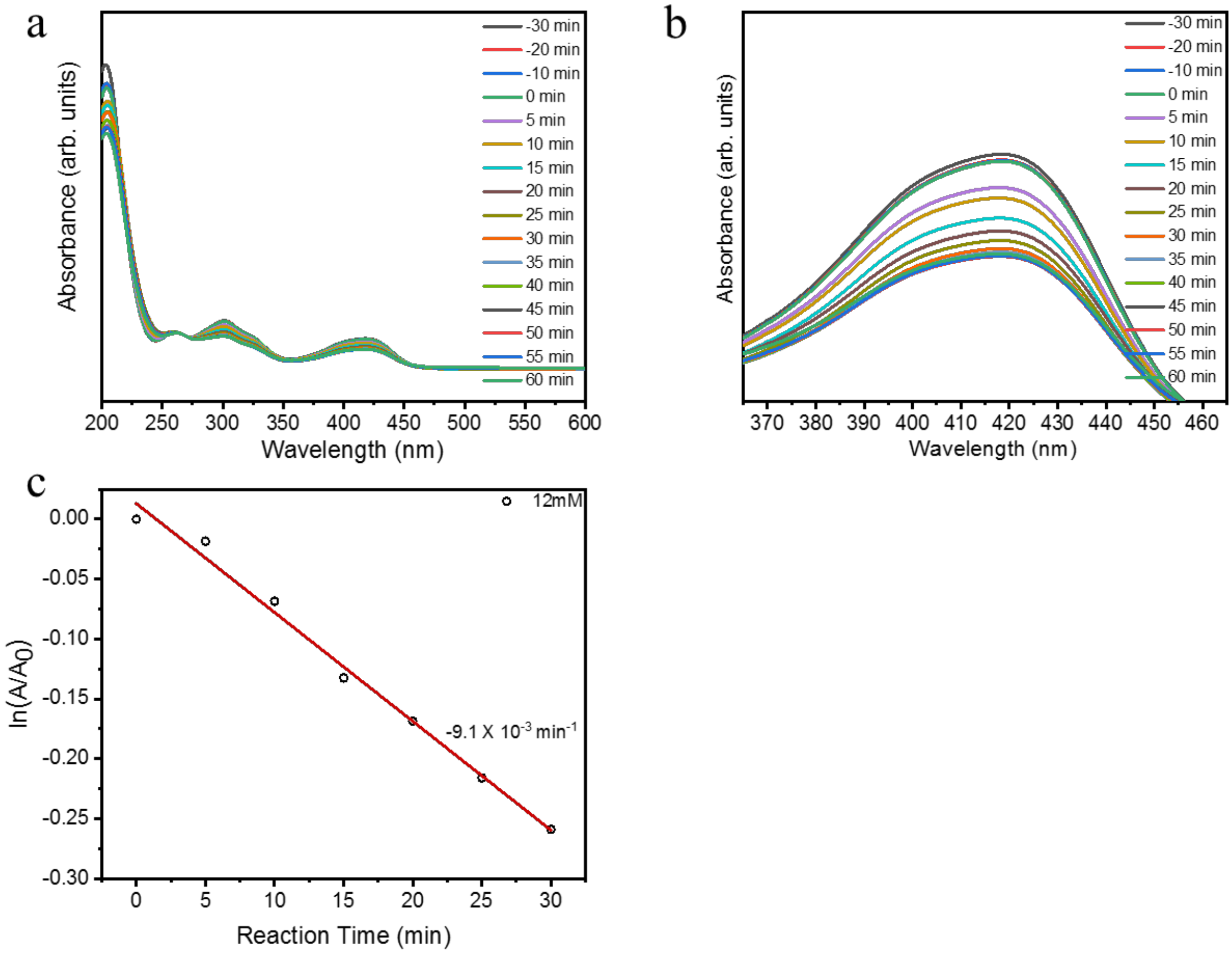

Figure S25. (a) UV-Vis spectra showing the conversion of Fe^{3+} to Fe^{2+} as a function of irradiation time for Ni_3N using 12 mM $K_3[Fe(CN)_6]$. (b) Magnified UV-Vis spectra around 419 nm. (c) Pseudo-first-order plot of $\ln(A/A_0)$ against reaction time for Ni_3N.

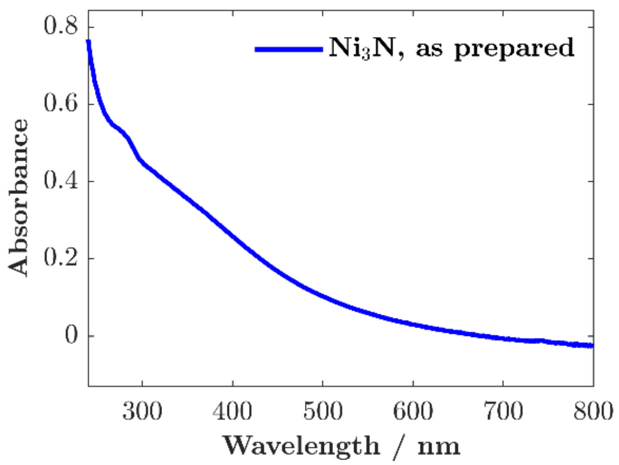

Figure S26. Absorption spectra of sonicated Ni_3N suspensions in ethanol, as-synthesized sample (left) and sample heated at 300 ºC (right).

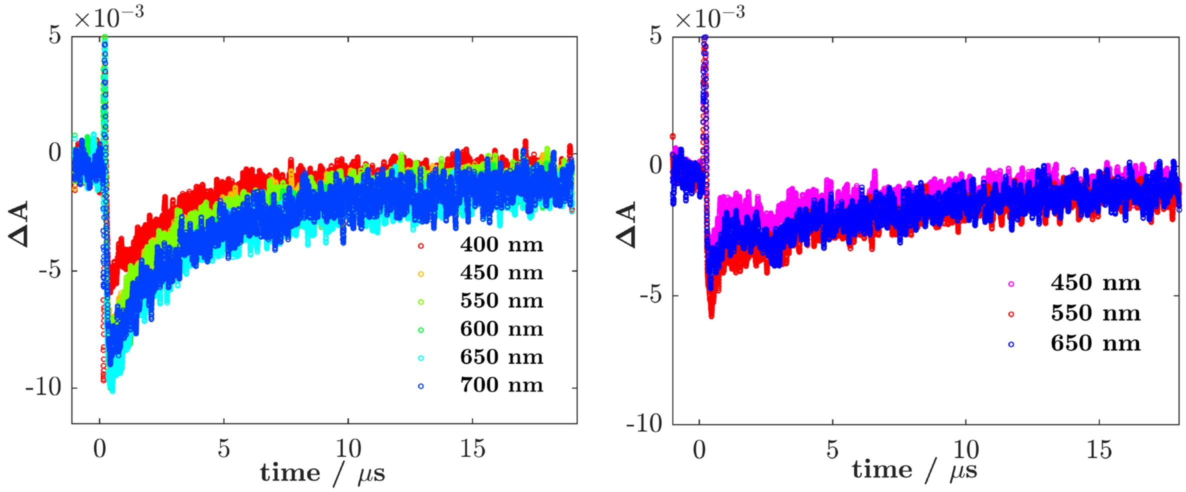

Figure S27. *Left:* Kinetic traces monitored at the indicated wavelengths in the legend for the as-synthesized Ni_3N sample, photoexcited at 500 nm (power=10 mJ/pulse). *Right:* Kinetic traces monitored at the indicated wavelengths in the legend for the as-synthesized Ni_3N sample saturated with CO_2, photoexcited at 500 nm (power=10 mJ/pulse). The observed dynamics are the same in both cases.

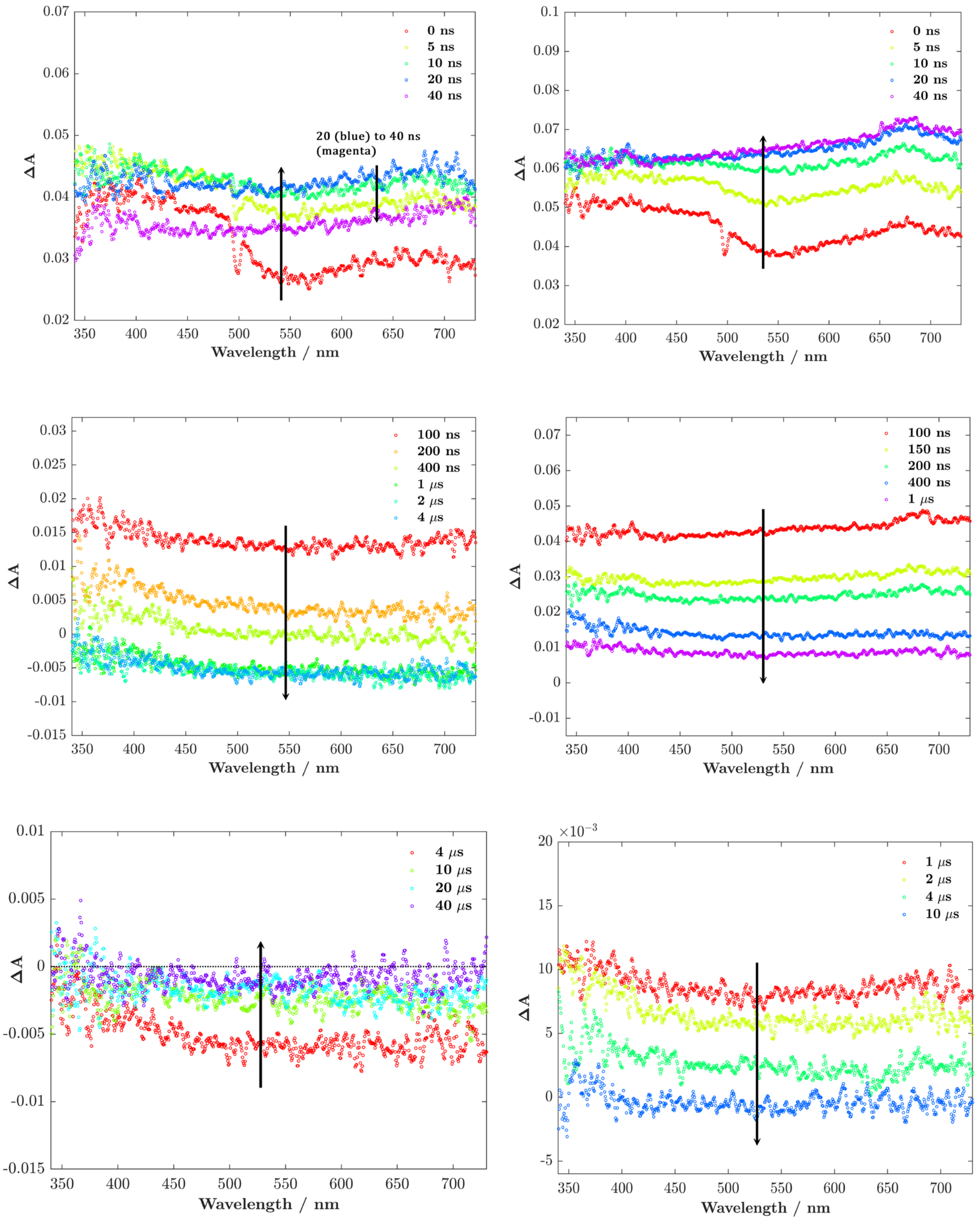

Figure S28. *Left column:* Spectra monitored at the indicated times in the legend for the heated Ni_3N sample, photoexcited at 500 nm (power=10 mJ/pulse). *Right column:* Spectra monitored at the indicated times in the legend for the heated Ni_3N sample saturated with CO_2, photoexcited at 500 nm (power=10 mJ/pulse). Note how the latter is characterized by a l arger positive offset at the beginning (the first row, left and right).

Supplementary Note 1. The calculation of the quantum efficiency of the photocatalytic CO_2 hydrogenation process

Quantum Efficiency is defined as:

$$Quantum\ Efficienccy\ (\%) = \frac{Produced\ CO\ molecules\ per\ unit\ time}{Absorbed\ photon\ numbers\ per\ unit\ time} \times 100$$

The no. of absorbed photons per unit time, N_{photon} is calculated from the emission spectra of Xe lamp and absorption spectra of Ni_3N.

$$N_{photon} = \sum_{\lambda=400\ nm}^{1100\ nm} \frac{Light\ Intensity\ \times\ I\%\ \times\ A\%\ \times Illumination\ area\ \times time}{Average\ single\ photon\ energy\ (E_{photon})\ \times\ N_A}$$

In photocatalytic CO_2 hydrogenation by Ni_3N, light intensity was varied on 1 cm^2 area. I% is the percentage of the light intensity of Xe-lamp at a specific wavelength and A% is the absorption percentage of the catalyst at that specific wavelength. Time is 1 h (3600 sec) and N_A is the Avogadro constant.

The average single photon energy is given by:

$$E_{photon} = \frac{hc}{\lambda}$$

In this equation, h is the Plank constant 6.626×10^{-34} m^2 kg s^{-1}, c is the speed of the light in a vacuum (3×10^8 m s^{-1}), and λ (meter) is the wavelength of the photon.

Table S1. Comparison of Photocatalytic $CO_2 + H_2$ reaction using both Plasmonic and Semiconducting Photocatalysts.

S. No.	Catalyst	Photocatalytic Conditions	Reactor Type	Temperature	Product Yield (mmol g_{cat}^{-1} h^{-1})	Product Selectivity (%)	Ref.
1	Ni_3N Nanosheets	P: 1 atm, L: Xenon lamp, 400-1100 nm, 3.006 W/cm^2	Flow	199 °C	CO : 1212<br>CH_4 : 0	99<br>0	This work
2	Ni@p-SiO_2	P: 1 atm, L: Xenon lamp, 2.8 W/cm^2	Flow	550°C	CO: 2100<br>CH_4: 1400	60<br>40	3
3	$Ni_{12}P_5/SiO_2$	P: 1 atm, L: Xenon lamp, no filter 0.8 W/cm^2	Flow	not given	CO : 13.5<br>CH_4 : NR	99.8<br>0.2	4
4	Nb_2C/Ni	P: 1 atm, L: Xenon lamp, 1.5 W/cm^2	Batch	300 °C	CO : 250<br>CH_4 : 1250	16.6<br>83.4	5
5	In_2O_{3-x}	P: 30 psi, L: Xenon lamp, 2 W/cm^2	Batch	300 °C	CO : 238.8	100	6
6	Au/TiO_2	P: 3.5 bar, L: Solar simulator, AM 1.5 1.44 W/cm^2	Batch	150°C	CO : 160<br>CH_4 : 10.5	93.8<br>6.2	7
7	Au/TiO_2	P: 110 psi, L: Visible light, 0.5216 W/cm^2	Batch	400°C	CO : 159	100	8
8	Cu_2O	P: 15 psi, L: Xenon lamp, 4 W/cm^2	Batch	322°C	CO : 70.3	100	9
9	Fe@C	P: 0.7 atm, L: Xenon lamp	Batch	450 °C	CO : 26.1	100	10
10	Ni/N-CeO_2	P: 1 atm, L: Xenon lamp	Flow	350 °C	CO : 20.9<br>CH_4 : 0	100<br>0	11

11	FeO–CeO_2	P: 1.77 atm, L: Xenon lamp, 2.2 W/cm^2	Flow	~ 450 °C	CO : 20	97	12
12	Rh/Al Nanoantenna	P: 1 atm, L: Solar simulator, 11.3 W/cm^2	Batch	200°C	CO : 15<br>CH_4 : 550	3<br>97	13
13	Au/TiO_2	P: 1 atm, L: Hg lamp, UV light, 0.150 W/cm^2	Flow	100°C	CO : 4.144<br>CH_4:0.000006	~ 100	14
14	2% Ag–0.5% Au/TNWs	P: 0.2 bar, L: Xenon lamp, 0.150 W/cm^2	Batch	not given	CO : 1.813<br>CH_4: 0.035	97.7<br>1.86	15
15	Pd@Nb_2O_5	P: 1 atm, L: Xenon lamp, 2.1 W/cm^2	Flow	~ 470 °C	CO : 1.8	100	16
16	Rh/Al_2O_3	P: 1 atm, L: UV LED (365 nm), 3 W/cm^2	Flow	350°C	CO : 1.8<br>CH_4: 21.6	7.7<br>92.3	17
17	Ag-NPs/TiO_2 NWs	P: 1 atm, L: Hg lamp, visible light, 20 mW/cm^2	Flow	100°C	CO : 0.983<br>CH_4: 0.00973<br>CH_3OH:0.013	~ 98	18
18	Al@Cu_2O	P: 1 atm, L: Supercontinuum laser, 400-859 nm, 10 W/cm^2	Flow	160°C	CO : 0.36	100	19
19	Ni/$SiO_2 \cdot Al_2O_3$	P: 1 atm, L: Solar simulator	Batch	150°C	CO : 0.392<br>CH_4 : 13.64	2.8<br>97.2	20
20	$In_2O_{3-x}(OH)_y$ nanorod	P: 14.1 psi, L: Xenon lamp, 160 mW/cm^2	Flow	200°C	CO : 0.2462	100	21
21	Ni-Au/SiO_2	P: 1 atm, L: Laser (520 nm), 684 W/cm^2	Flow	450°C	CO : 0.18<br>CH_4: 0.16	53<br>47	22

22	$Pt/NaTaO_3$	P: 40 kPa, L: Xenon lamp, 2 W/cm^2	Batch	not given	CO : 0.134	99	23
23	Hydride-terminated nano-silicon	P: 27 psi, L: Xenon lamp, 1.5 W/cm^2	Batch	150 °C	CO : 0.083<br>CH_4 : 0	100<br>0	24
24	$Cu/Pd/HyWO_{3-x}$	P: 1 atm, L: Xenon lamp, 2 W/cm^2	Flow	250 °C	CO : 0.041	100	25
25	Palladium-decorated silicon–hydride	P: 27 psi, L: Xenon lamp, 1.5 W/cm^2	Batch	170°C	CO : 0.00001<br>CH_4 : 0	0<br>100	26
26	FL-LDHs/Ru	P: 1 atm, L: Xenon lamp, 1 W/cm^2	Flow	350 °C	CO : 0<br>CH_4 : 40	0<br>100	27
27	RuO_2 on silicon photonic crystal	P: 1 atm, L: Xenon lamp, 2.2 W/cm^2	Flow	150°C	CO : 0<br>CH_4 : 4	0<br>100	28
28	Ni-BTO	P: 4 atm, L: Xenon lamp, 0.293 W/cm^2	Batch	350 °C	CO : 0<br>CH_4 : 103.7	0<br>100	29
29	Ru/Silicon Nanowire	P: 15 psi, L: Xenon lamp, 1.45 W/cm^2	Flow	93 °C	CO : 0<br>CH_4 : 0.74	0<br>100	30
30	Graphene Supp. NiO/Ni	P: 1.3 atm, L: Xenon lamp, 0.2236 W/cm^2	Batch	200°C	CO : 0<br>CH_4 : 0.642	0<br>100	31
31	Ni/CeO_2	P: 1 atm, L: Xenon lamp, 0.5 W/cm^2	Flow	250°C	CO : 0<br>CH_4 : 2.5	0<br>100	32
32	Ti-IPA MOF	P: 1 atm, L: Xenon lamp, 0.23 W/cm^2,	Batch	200 °C	CO : 0<br>CH_4 : 0.033	0<br>100	33

33	RuO_2/STO	P: 1.3 bar, L: Xenon lamp, 0.108 W/cm^2	Batch	150°C	CO : 0<br>CH_4 : 14.6	0<br>100	34
34	Ag_{25} Clusters	P: 1 atm, L: Xenon lamp, 420-780, 0.183 W/cm^2	Batch	100°C	CO : 0<br>CH_4 : 0.029	0<br>100	35
35	Ni/Al_2O_3·SiO_2	P: 1 atm, L: LED lamp, 2.2 W/cm^2	Batch	225°C	CO : 0<br>CH_4: 35	0<br>100	36
36	Rh/TiO_2	P: 1 atm, L: UV LED (365 nm), 2.73 W/cm^2	Flow	282°C	CO : 0<br>CH_4: 0.028	0<br>100	37
37	SA Ni/Y_2O_3	P: 1 atm, L: Xenon lamp, 2 W/cm^2	Flow	~ 300 °C	CO : 0<br>CH_4: 7.5 L m^{-2} h^{-1}	0<br>100	38

* *Pressure (P), Light Source (L), Temperature (T), Reactor (R),. In unit mmol g_{cat}^{-1} h^{-1}, $_{cat}$ is active site amount.*

Supplementary References

1. N. Kaul, R. Lomoth. The carbene cannibal: Photoinduced symmetry-breaking charge separation in an Fe(III) *N*-heterocyclic carbene. *J. Am. Chem. Soc.* **143**, 10816–10821 (2021).
2. A. Baiker , M. Maciejewski. Formation and thermal stability of copper and nickel nitrides. *J. Chem. Soc. Faraday Trans. I* , **80**, 2331-2341 (1984).
3. Cai, M.; Wu, Z.; Li, Z.; Wang, L.; Sun, W.; Tountas, A. A.; Li, C.; Wang, S.; Feng, K.; Xu, A. B.; Tang, S.; Tavasoli, A.; Peng, M.; Liu, W.; Helmey, A. S.; He L.; Ozin, G. A.; Zhang X. Greenhouse-inspired supra-photothermal CO_2 catalysis. *Nat. Energy*, **6**, 807-814 (2021).
4. Xu, Y. -F., Duchesne, P. N., Wang, L., Tavasoli, A., Jelle, A. A., Xia, M., Liao, J. -F., Kuang, D. -B., Ozin, G. A. High-performance light-driven heterogeneous CO_2 catalysis with near-unity selectivity on metal phosphides. *Nat. Commun.* **11**, 5149 (2020).
5. Wu, Z., Li, C., Li, Z., Feng, K., Cai, M., Zhang, D., Wang, S., Chu, M., Zhang, C., Shen, J., Huang, Z., Xiao, Y., Ozin, G. A., Zhang, X., He, L. Niobium and titanium carbides (mxenes) as superior photothermal supports for CO_2 photocatalysis. *ACS Nano* **15**, 5696−5705 (2021).
6. Wang, L. et al. Black indium oxide a photothermal CO2 hydrogenation catalyst. *Nat. Commun.* **11**, 2432 (2020)
7. Molina, P. M., Meulendijks, N., Xu, M., Verheijen, M. A., den Hartog, T., Buskens, P., Sastre, F. Low temperature sunlight-powered reduction of CO_2 to CO using a plasmonic Au/TiO_2 nanocatalyst. *ChemCatChem* **13**, 4507–4513 (2021).
8. Upadhye, A. A., Insoo Roa , Zenga, X., Kim, H. J., Tejedor, I., Anderson, M. A., Dumesic J. A., Hub, G. W. Plasmon-enhanced reverse water gas shift reaction over oxide supported Au catalysts. *Catal. Sci. Technol.,* **5**, 2590-2601 (2015).
9. Wan, L. L. et al. Cu_2O nanocubes with mixed oxidation-state facets for (photo)catalytic hydrogenation of carbon dioxide. *Nat. Catal.* **2**, 889-898 (2019).
10. Zhang, H. et al. Surface-Plasmon-Enhanced Photodriven CO_2 Reduction Catalyzed by Metal-Organic-Framework-Derived Iron Nanoparticles Encapsulated by Ultrathin Carbon Layers. *Adv. Mater.* **28**, 3703-3710 (2016).
11. Jia, Z., Ning, S., Tong, Y., Chen, X., Hu, H., Liu, L., Ye, J., Wang, D. Selective photothermal reduction of CO_2 to CO over Ni-nanoparticle/N-doped CeO_2 nanocomposite catalysts. *ACS Appl. Nano Mater.* **4**, 10485−10494 (2021).
12. Zhao, J. et al. FeO–CeO2 nanocomposites: an efficient and highly selective catalyst system for photothermal CO_2 reduction to CO. *NPG Asia Mater.* **12**, 5 (2020).
13. Fu, G., Jiang, M., Liu, J., Zhang, K., Hu, Y., Xiong, Y., Tao, A., Tie, Z., Jin, Z. Rh/Al Nanoantenna photothermal catalyst for wide-spectrum solar-driven CO_2 methanation with nearly 100% selectivity. *Nano Lett.* **21**, 8824−8830 (2021).
14. Tahir, B., Tahir, M., Amin, N. A. S. Photocatalytic CO_2 conversion over Au/TiO_2 nanostructures for dynamic production of clean fuels in a monolith photoreactor. *Clean Techn. Environ. Policy*, doi 10.1007/s10098-016-1181-4 (2016).
15. Tahir, M., Tahir, B., Amin, N. A. S. Synergistic effect in plasmonic Au/Ag alloy NPs co-coated TiO_2 NWs toward visible-light enhanced CO_2 photoreduction to fuels. *Appl. Catal. B: Environ.* **204**, 548–560 (2017).
16. Jia, J. et al. Photothermal catalyst engineering: hydrogenation of gaseous CO_2 with high activity and tailored selectivity. *Adv. Sci.* **4**, 1700252 (2017).
17. Zhang, X.; Li, X.; Zhang, D.; Su, Q. N.; Yang, W.; Everitt, O. H.; Liu, J. Product selectivity in plasmonic photocatalysis for carbon dioxide hydrogenation. *Nat Commun*, **8**, 14542 (2017).
18. Tahir, M., Tahir, B., Amin, N. A. S., Zakaria, Z. Y. Photo-induced reduction of CO_2 to CO with hydrogen over plasmonic Ag-NPs/TiO_2 NWs core/shell hetero-junction under UV and visible light. *J. CO_2 Util.* **18**, 250–260 (2017).
19. Robatjazi, H., Zhao, H., Swearer, D. F., Hogan, N. J., Zhou, L., Alabastri, A., McClain, M. J., Nordlander, P., Halas, N. J. Plasmon-induced selective carbon dioxide conversion on earth-abundant aluminum-cuprous oxide antenna-reactor nanoparticles. *Nat Commun*, **8**, 27 (2017).
20. Sastre, F., Puga, A. V., Liu, L., Corma, A., García, H. Complete photocatalytic reduction of CO_2 to methane by H_2 under solar light irradiation. *J. Am. Chem. Soc.* **136**, 6798−6801 (2014).
21. Loh, J. Y. Y., Mohan, A., Flood, A. G., Ozin, G. A., Kherani, N. P. Waveguide photoreactor enhances solar fuels photon utilization towards maximal optoelectronic – photocatalytic synergy. *Nat Commun*, **12**, 402 (2021).

22. J. N. G., García-García, I., Perfrement, T., Lovell, E. C., Schmidt, T. W., Scott, J., Amal, R. Plasmonic effects on CO_2 reduction over bimetallic Ni-Au catalysts. Stanley. *Chem. Eng. Sci.* **194**, 94-104 (2019).
23. Li, M. et al. Highly efficient and stable photocatalytic reduction of CO_2 to CH_4 over Ru loaded $NaTaO_3$. *Chem. Commun.* **51**, 7645-7648 (2015).
24. Sun, W., Qian, C., He, L., Ghuman, K. K., Wong, A.P.Y., Jia, J., Jelle, A. A., O'Brien, P. G., Reyes, L. M., Wood, T. E., Helmy, A. S., Mims, C. A., Chandra Veer Singh, C. V., Ozin, G. A. Heterogeneous reduction of carbon dioxide by hydride-terminated silicon nanocrystals. *Nat. Commun*. **7**, 12553 (2016).
25. Li, Y. F. et al. Cu atoms on nanowire Pd/HyWO3–x bronzes enhance the solar reverse water gas shift reaction. *J. Am. Chem. Soc.* **141**, 14991-14996 (2019).
26. Qian, C., Sun, W., Hung, D. L.H., Qiu, C., Makaremi, M., Kumar, S. G. H., Wan, L., Ghoussoub, M., Wood, T. E., Xia, M., Tountas, A. A., Li, Y. F., Wang, L., Dong, Y., Gourevich, I., Singh, C. V., Ozin, G. A. Catalytic CO_2 reduction by palladium-decorated silicon–hydride nanosheets. *Nat. Catal.* **2**, 46–54 (2019).
27. Ren, J., Ouyang, S., Xu, H., Meng, X., Wang, T., Wang, D., Jinhua Ye, J. Targeting activation of CO_2 and H_2 over Ru-loaded ultrathin layered double hydroxides to achieve efficient photothermal CO_2 methanation in flow-type system. *Adv. Energy Mater.* **7**, 1601657 (2017).
28. Jelle, A. A., Ghuman, K. K., O'Brien, P. G., Hmadeh, M., Sandhel, A., Perovic, D. D., Singh, C. V., Mims, C. A., Ozin, G. A. Highly efficient ambient temperature CO_2 photomethanation catalyzed by nanostructured RuO_2 on silicon photonic crystal support. *Adv. Energy Mater.* **8**, 1702277 (2018).
29. Mateo, D., Morlanes, N., Maity, P., Shterk, G., Mohammed, O. F., Jorge Gascon, J., Efficient visible-light driven photothermal conversion of CO_2 to methane by nickel nanoparticles supported on barium titanate. *Adv. Funct. Mater.* **31**, 2008244 (2021).
30. O'Brien, P. G., Sandhel, A., Wood, T. E., Jelle, A. A., Hoch, L. B., Perovic, D. D., Mims, C. A., Ozin, G. A. Photomethanation of gaseous CO_2 over Ru/silicon nanowire catalysts with visible and near-infrared photons. *Adv. Sci.* **1**, 1400001 (2014).
31. Mateo, D., Albero, J., García, H. Graphene supported NiO/Ni nanoparticles as efficient photocatalyst for gas phase CO_2 reduction with hydrogen. *Appl. Catal. B: Environ.* **224**, 563–571 (2018).
32. Golovanova, V., Spadaro, M. C., Arbiol, J., Golovanov, V., Rantala, T. T., Andreu, T., Morante, J. R. Effects of solar irradiation on thermally driven CO_2 methanation using Ni/CeO_2–based catalyst. *Appl. Catal. B: Environ.* **291** 120038 (2021).
33. Wang, S., Cabrero-Antonino, M., Navalón, S., Cao, C. -C., Tissot, A., Dovgaliuk, I., Marrot, J., Martineau-Corcos, C., Yu, L., Wang, H., Shepard, W., Garcia, H., Serre, C. A robust titanium isophthalate metal-organic framework for visible-light photocatalytic CO_2 methanation. *Chem* **6**, 3409–3427 (2020).
34. Mateo, D., Albero, J., Garcia, H. Titanium-perovskite-supported RuO_2 nanoparticles for photocatalytic CO_2 methanation. *Joule* **3**, 1949–1962 (2019)
35. Xiong, Y., Chen, H., Hu, Y., Yang, S., Xue, X., He, L., Liu, X., Ma, J., Jin, Z. photodriven catalytic hydrogenation of CO_2 to CH_4 with nearly 100% selectivity over Ag_{25} clusters. *Nano Lett.* **21**, 8693−8700 (2021).
36. Bueno-Alejo, C. J., Arca-Ramos, A., Hueso, J. L. Santamaria, J. LED-driven continuous flow carbon dioxide hydrogenation on a nickel-based catalyst. *Catal. Today* **355**, 678-684 (2020).
37. Li, X.; Everitt, O. H.; Liu, J. Confirming nonthermal plasmonic effects enhance CO_2 methanation on Rh/TiO_2 catalysts. *Nano Res.* **2019**, 12, 1906–1911.
38. Li, Y. et al. Selective light absorber-assisted single nickel atom catalysts for ambient sunlight-driven CO_2 methanation. *Nat. Commun.* **10**, 2359 (2019).
