# Supplementary material for: Surface plasmon-enhanced photo-driven CO2 hydrogenation by hydroxy-terminated nickel nitride nanosheets
Source: Nat Commun. 2023 May 3;14:2551. doi: 10.1038/s41467-023-38235-9 (PMC10156734; doi:10.1038/s41467-023-38235-9)
Supplement: Supplementary file 2 — Description of Additional Supplementary Files [file 41467_2023_38235_MOESM2_ESM.pdf]

Description of Additional Supplementary Files

File Name: Supplementary Data 1
Description: Detailed calculations of CO productivity

File Name: Supplementary Data 2
Description: Detailed calculations of quantum efficiency as a function of intensity at various reaction temperatures.
